# LncRNAs in Ovarian Cancer Progression, Metastasis, and Main Pathways: ceRNA and Alternative Mechanisms

**DOI:** 10.3390/ijms21228855

**Published:** 2020-11-23

**Authors:** Eleonora A. Braga, Marina V. Fridman, Alexey A. Moscovtsev, Elena A. Filippova, Alexey A. Dmitriev, Nikolay E. Kushlinskii

**Affiliations:** 1 Institute of General Pathology and Pathophysiology, 125315 Moscow, Russia; alexey.moscovtsev@gmail.com (A.A.M.); p.lenyxa@yandex.ru (E.A.F.); 2 Research Centre for Medical Genetics, 115522 Moscow, Russia; 3 Vavilov Institute of General Genetics, Russian Academy of Sciences, 119991 Moscow, Russia; marina-free@mail.ru; 4 Engelhardt Institute of Molecular Biology, Russian Academy of Sciences, 119991 Moscow, Russia; alex_245@mail.ru; 5 N.N. Blokhin National Medical Research Center of Oncology, 115478 Moscow, Russia; kne3108@gmail.com

**Keywords:** ovarian cancer, long non-coding RNAs, competitive endogenous RNAs, miRNAs, oncogenic and suppressive lncRNAs, metastasis, signaling pathways

## Abstract

Ovarian cancer (OvCa) develops asymptomatically until it reaches the advanced stages with metastasis, chemoresistance, and poor prognosis. Our review focuses on the analysis of regulatory long non-coding RNAs (lncRNAs) competing with protein-coding mRNAs for binding to miRNAs according to the model of competitive endogenous RNA (ceRNA) in OvCa. Analysis of publications showed that most lncRNAs acting as ceRNAs participate in OvCa progression: migration, invasion, epithelial-mesenchymal transition (EMT), and metastasis. More than 30 lncRNAs turned out to be predictors of survival and/or response to therapy in patients with OvCa. For a number of oncogenic (CCAT1, HOTAIR, NEAT1, and TUG1 among others) and some suppressive lncRNAs, several lncRNA/miRNA/mRNA axes were identified, which revealed various functions for each of them. Our review also considers examples of alternative mechanisms of actions for lncRNAs besides being ceRNAs, including binding directly to mRNA or protein, and some of them (DANCR, GAS5, MALAT1, and UCA1 among others) act by both mechanisms depending on the target protein. A systematic analysis based on the data from literature and Panther or KEGG (Kyoto Encyclopedia of Genes and Genomes) databases showed that a significant part of lncRNAs affects the key pathways involved in OvCa metastasis, EMT, and chemoresistance.

## 1. Introduction

In the last two decades, the important role of non-coding RNAs (ncRNAs) in the regulation of gene expression and signaling pathways has been established [1,2]. By analyzing genomic databases, evidence has been obtained that the number of ncRNA genes in eukaryotes, including humans, is many times greater than the number of known and predicted protein-encoding genes, and thousands of new functional ncRNAs have been discovered. ncRNAs can be conventionally classified by length: long (from about 200 to tens of thousands of nucleotides) and short (20–200 nucleotides) [3].

Among short RNAs, the most studied are microRNAs (miRNAs), for which a key role in the regulation of gene expression has been established. Despite the fact that usually the regulatory action of miRNAs is very “subtle”, as it changes the expression of the target gene by no more than two-fold [4], the Encyclopedia of DNA elements (ENCODE) consortium placed miRNAs at the beginning of regulatory networks, assigning them the role of “master regulators” of signaling cascades in the cell [5]. It is believed that miRNAs control the expression of about 50% of protein-coding genes, from which it follows that they regulate many vital processes of cell activity: proliferation, differentiation, apoptosis, adhesion, epithelial-mesenchymal transition (EMT), and metastasis, which is supported by studies of tumors of different origin [6,7]. In recent years, the regulation processes of miRNAs themselves have become topical, in particular the effect of promoter methylation on miRNA gene expression [8] and the role of recently discovered long ncRNA (lncRNA) and circular ncRNA [9,10].

With the use of new-generation sequencing technologies, it turned out that most genomic sequences are transcribed in the form of lncRNAs, which surpass 100 thousand (see databases http://www.noncode.org and http://www.lncrnadb.org/) and exceed the count of known protein-coding genes and identified human miRNAs [11]. LncRNA molecules do not encode proteins, since they do not have an open reading frame of sufficient length (although exceptions have been described when a protein can still be encoded with RNA itself playing a significant role [12]). As a rule, the evolutionary conservation of the entire lncRNA sequence is significantly less than that of genes encoding proteins but higher than that of non-transcribed regions. Moreover, the conservation of the promoter regions of lncRNA genes is the same as in genes encoding proteins, and the expression of lncRNA directed by them is highly tissue-specific [13].

The lncRNA molecules are more than 200 bases in length, are transcribed by RNA polymerase II, and capped and polyadenylated at the 5’ and 3’ ends, respectively [13]. The sequences encoding lncRNAs can be located in intergenic regions, in introns, or partially overlapping exons localized both on the forward and reverse strands. As a result, they can be divided into five subclasses: sense, antisense, bidirectional, intergenic, and intronic.

LncRNAs are involved in a variety of processes: from the modification of histones and chromatin remodeling to the regulation of transcriptional and posttranscriptional processes. They can be enhancers, scaffolds, “sponges” that bind several miRNAs, or even precursors of some miRNAs [9]. Aberrant expression of lncRNA, in particular oncogenic lncRNA, leads to disturbances in cell signaling cascades, which can affect cell proliferation and promote tumor progression and metastasis [14]. For example, lncRNA DANCR (differentiation antagonizing non-protein coding RNA) generally works as a tumor promoter by binding with corresponding miRNAs or by interacting with various regulating proteins [15].

Currently, the most widely accepted model of competitive endogenous RNAs (ceRNAs) describes lncRNAs competing with protein-coding mRNAs for binding to miRNAs. It was found that lncRNAs interact with the same miRNA segments that are involved in the binding of mRNA-targets. So, back in 2011, Salmena et al. [16] put forward a hypothesis according to which transcripts of protein-coding genes, pseudogenes, miRNAs, and lncRNAs are involved in a complex network of interactions owing to miRNA response elements. However, direct lncRNA interactions with protein or mRNA are also observed, for example, WDR7-7 lncRNA with mRNA of the *GPR30* gene [17]. Moreover, large-scale regulatory networks have already been outlined for lncRNA, mRNA, and miRNA in cancer cells [18,19,20]. LncRNAs add new layers to regulatory networks and deepen our understanding of the mechanisms of oncogenesis, which is necessary for a constructive approach to diagnosis, prognosis on cancer progression, response to therapy, as well as for the search for new therapeutic drugs. The clinical significance of lncRNAs as diagnostic and prognostic markers and therapeutic targets in various types of cancer, including ovarian cancer, has been shown [21,22,23].

Ovarian cancer (OvCa) belongs to the insidious, most late detected types of cancer. It develops asymptomatically up to advanced stages and is characterized by extensive and most commonly abdominal metastasis, with ascites formation, chemoresistance, and poor prognosis. In 2018, about 295,000 new cases and 185,000 deaths around the world were counted, with increasing trends predicted [24]. OvCa remains the deadliest worldwide cancer of the female reproductive tract [25]. Despite recent improvements in cytoreductive surgery and chemotherapy, its five-year overall survival rate is less than 30%, owing to its late diagnosis at an advanced stage with metastasis and chemotherapy resistance [24,26]. Many patients with advanced disease will undergo recurrence within 12–18 months.

Thus, there is an urgent need not only to search for new effective diagnostic and prognostic markers and to develop programs for screening patients with OvCa but also to determine effective markers of response to treatment. This will allow timely detection of the disease, prediction of its course, and evaluation of treatment effectiveness. All this is possible when solving the key issue—elucidating the molecular mechanisms of gene regulation and signaling pathways in the pathogenesis and metastasis of OvCa, including the role of ncRNA in these processes.

This review collects and summarizes the literature data accumulated over the past few years on the participation of lncRNAs in gene regulation through the ceRNA mechanism, as in through the lncRNA/miRNA/mRNA axis, in the pathogenesis, progression, and metastasis of OvCa. This section of the overview is preceded by information on unresolved issues around the ceRNA hypothesis. Further, the main signaling pathways in OvCa and metastasis also precede consideration of lncRNA acting through the ceRNA mechanism. In the last sections, examples of alternative mechanisms of gene regulation involving lncRNAs in OvCa and the effect of lncRNAs on signaling pathways in metastasis and drug resistance are discussed.

## 2. ceRNA Hypothesis and New Methods for Its Unresolved Questions

The cornerstone of the functional activity of long non-coding RNAs is their proposed role in the novel model of competitive endogenous RNA (ceRNA). This hypothesis attracts attention since it considers the different classes of RNA interacting with each other. This interaction is mediated by the RNA-induced silencing complex (RISC) and determines the level of post-transcriptional regulation of gene expression. In our opinion, the ceRNA concept is interesting primarily as a mechanism for the integration of information in the cell; indeed, thanks to RNA–RNA interactions, information flows transcribed from different genomic loci within the framework of different functional activities can be summarized not only in the nucleus but also in the cytoplasm. Cytoplasmic RNA-mediated information integration can be of great importance since it occurs just before translation and is thus one of the last regulation steps before RNA decoding into protein.

Due to the growing potential to consider the ceRNA principle as a universal mechanism acting in the cells, it is necessary to check its quantitative foundations, which should be based on the complex kinetics of thousands of ribonucleoproteins-RNA interactions.

Denzler et al. [27] and Bosson et al. [28] have attempted to assess the ceRNA hypothesis by quantifying the number of binding sites/miRNA response elements (MREs) that must be introduced into the cell to detect ceRNA-mediated gene regulation. These studies agree that quantifying miRNA binding sites in the transcriptome is critical to assessing the regulatory potential of ceRNA and that the number of miRNA binding sites is generally higher than the number of miRNA molecules in the cell [27].

Bosson et al. [28] suggest that the enrichment of certain miRNA in the cell determines its susceptibility to ceRNA-mediated regulation. When the concentration of certain miRNA increases, its complexes with Argonaute (AGO) redistribute to low-affinity sites (8 nt > 7 nt > 6 nt sites) [28]. For highly expressed miRNA, the enrichment of the effective target sites is too large for the physiological ranges of ceRNA expression to influence repression. Physiological changes in ceRNA may nevertheless influence repression for low or moderately expressed miRNAs with a lower number of target sites [28].

Denzler et al. [27] suggest that sites of all affinities contribute to an effective amount of targets, regardless of miRNA concentration. The de-repression threshold remains constant when miRNA activity is reduced [27]. Low-affinity sites, due to their large number in the transcriptome, make a significant total contribution to the effective number of target sites for each miRNA—even for more moderately expressed miRNAs [27]. Since the number of targets for a typical miRNA is very large, the regulation of gene expression by a single ceRNA is unlikely to occur in differentiated cells under physiological conditions and even in pathology [27]. For that regulation, ceRNA expression should reach extreme values comparable with the total amount of targets. In addition, a ceRNA must have at least several MREs for certain miRNA. However, lncRNAs are often characterized by low expression levels and a small number of MREs per miRNA family.

In addition, there are still a number of unresolved fundamental problems around the ceRNA hypothesis. The “rules” of the interaction of miRNA with target RNA are not clear enough yet. Despite the current abundance of data from the analysis of direct RNA–RNA association, binding affinities of miRNA-induced silencing complex (miRISC) with the target have not been fully established due to sparsity of the data—a small number of analyzed motifs. In the recent work, a high-throughput analysis of an artificially synthesized library of all variants of 12-nucleotide sequences, coupled with an improved RNA bind-n-seq method for the extraction of AGO-miRNA complexes, refined the rules of miRNA-target binding and increased the target prediction accuracy of the model to 60% [29]. In that study, the affinities of the AGO-miRNA complexes were determined for several miRNAs. Moreover, non-canonical sites and a 100-fold effect of dinucleotides flanking MRE on affinity were established [29].

When assessing ceRNA and RISC functional activity, the assumptions are often made on the uniform distribution of interacting molecules in the volume of the solvent. Wherein, the significant heterogeneity in the cell is ignored. In particular, membraneless organelles, such as P bodies, stress, and other RNA granules, significantly change the reaction conditions: local concentration balance, the viscosity, the diffusion rate.

For characterizing lncRNA ceRNA-associated functionality, the methods assessing lncRNA physical binding interactome are of great importance. Specifically, the analysis of direct RNA–RNA association, initially based on approaches for detecting RNA–protein interactions such as RNA immunoprecipitation (RIP), has recently evolved towards increasing the specificity of the determined interactions and unambiguous mapping of their sites on RNA molecules (AGO HITS-CLIP, PAR-CLIP, CLASH, etc.). However, these methods of RNA–RNA association analysis mainly provide information about static snapshots of interactions. In this regard, we could expect a refinement of our knowledge about the dynamic mechanisms of target and MRE search by the RISC complex in the cell, e.g., the kinetics of the interaction of the AGO with the target in the artificial systems with immobilized RNA molecules was studied. The results obtained [30] allow the authors to suggest a mechanism for target identification by the miRISC complex, according to which AGO2 first binds to the target RNA and undergoes lateral diffusion along the RNA molecule by scanning it with sub-seed sequence (2nd–4th nucleotides). Complementarity can then extend to the entire seed sequence. The authors suggest that mechanism as the most effective one for scanning unstructured RNA regions, which reduces the search time. The spreading of complementarity from the sub-seed to the seed competes with the dissociation of the RISC complex and contributes to its retention on the target RNA [30]. In another study, it was shown that the RISC complex alters the thermodynamics of miRNA–RNA interaction so that the association Kon rate increases significantly, which shortens the target search time and brings it closer to the rate of macromolecular diffusion [31].

Along with the aforementioned studies, which question the ceRNA hypothesis, the number of existing methods for verifying the influence of lncRNA convincingly proves that under certain conditions, the effect of this type of interaction is still there. However, since the latest results speak in favor of the fact that the effect does not have to be everywhere and always, it is very important to analyze the functional activity of lncRNA in the specific cellular context.

Since theoretical predictions of effective interactions by the ceRNA mechanism are not sufficiently reliable at present, in this review, we consider primarily those works in which there is clear and complete experimental evidence of such an interaction (see below).

## 3. The Most Important Signaling Pathways in OvCa

Previously, our review work discussed in detail the processes and mechanisms involved in the pathogenesis and metastasis of OvCa [32]. In the next sections, we focused on the signaling pathways that are most significant in OvCa and metastasis.

According to the review [33], the lysophosphatidic acid signaling pathway is impaired in 90% of OvCa cases, the phosphoinositide 3-kinase (PI3K) signaling in 70% of cases, nuclear factor kappa-light-chain-enhancer of activated B cells (NF-κB) in 50%, while the contribution of the mitogen-activated protein kinase (MAPK) pathway disorders is slightly less than 50%. In the pathogenesis of OvCa, disturbances in the proto-oncogene tyrosine-protein kinase Src, receptor tyrosine kinase ErbB, Müllerian inhibiting substance receptor, epidermal growth factor (EGF), vascular endothelial growth factor (VEGF), Janus kinase/signal transducer and activator of transcription 3 (JAK/STAT3), interleukin-6/ interleukin-6 receptor (IL-6/IL-6R), and Janus kinase 2 signal transducer pathways can also play a role. Inherited mutations in *BRCA1* and *BRCA2* genes, mutations in genes of the DNA mismatch repair system (Lynch syndrome), and, to a lesser extent, mutations in *TP53*, *PTEN*, *CHEK2*, and *PALB2* genes predispose to OvCa.

Different types of ovarian cancer vary in their aggressiveness and morphological characteristics. The Cancer Genome Atlas group studied transcriptional activity in ovarian cancer samples and identified 4 subtypes of OvCa. According to their data, 67% of cases of the disease are also associated with disorders of the Rb pathway. In 96% of the most metastatically active poorly differentiated serous OvCa, *TP53* mutations are characteristic. The mesenchymal subtype of poorly differentiated serous cancer differs both in the specificity of the expression of structural genes (high expression of genes of the Wnt signaling pathway, genes of transcription factors that regulate ontogenesis, and *HOX* genes; decreased expression of E-cadherin) and in the specificity of miRNA expression [34].

Later, based on the analysis of expression data, copy number of different genes, and data on protein interactions, two subtypes of ovarian cancer were identified, and the second one with a significantly worse prognosis. The first subtype was characterized by the activation of the Ras pathway, the second—the violation of the Rb pathway, as well as an increase in the expression of genes of the VEGF pathway [35].

Currently, studies are ongoing to identify the key regulators of oncogenesis, but they generally confirm the current picture. Thus, the important role of the PI3K/protein kinase B (AKT)/mammalian target of rapamycin (mTOR) and NF-κB pathways is emphasized, as in OvCa they are often hyperactive and susceptible to mutations, which correlates with a poor prognosis [36]. Bioinformatics methods have revealed five key regulators in OvCa: *AKT1*, *KRAS*, *EPCAM*, *CD44*, and *MCAM*; *AKT1*, as one might expect, plays the most important role [37]. A separate significant point is the ratio of AKT pathways and autophagy. In general, the expression of many oncogenes (*AKT*, *BCL2*) reduces autophagy in OvCa; but some of them, for example, *RAS*, may act differently in varied conditions. In contrast, tumor suppressors (PTEN, TSC1/TSC2, and DAPK) stimulate autophagy [38]. ULK1 plays an important role in the autophagy process, being under the control of the kinases AMPK and mTOR. The PI3K/AKT signaling pathway inhibits autophagy by activating the mTOR-mTORC1 complex, which inhibits complexes with ULK1. This signaling pathway is activated by growth factors and inhibited during starvation [38]. PTEN, 3-phosphatase of phosphatidylinositol-3,4,5-phosphate, negatively regulates the PI3K/AKT/PKB signaling pathway. A high level of phosphorylation of AKT and mTOR (and, accordingly, a high level of activity) correlates in OvCa with a poor prognosis [39].

Recent results from laboratory and clinical studies predominantly clarify already known mechanisms. Thus, a recent review of the mechanisms associated with the regulation of apoptosis in OvCa and their use in therapy emphasizes the role of JAK/STAT, Wnt/β-catenin, MAPK/ERK, and PI3K/AKT/mTOR pathways [40].

Of particular interest are proteins and signaling pathways, whose roles differ at different stages of OvCa development. The most famous example is transforming growth factor beta (TGF-β), which suppresses this cancer in its early stages. At more advanced stages, genes that are normally activated by TGF-β become epigenetically repressed [41], and TGF-β is involved in the activation of the transcription factor SNAIL, which leads to EMT [41] and other oncogenic effects, for example, the significant activation of STAT1 [42,43].

**Figure 1:**
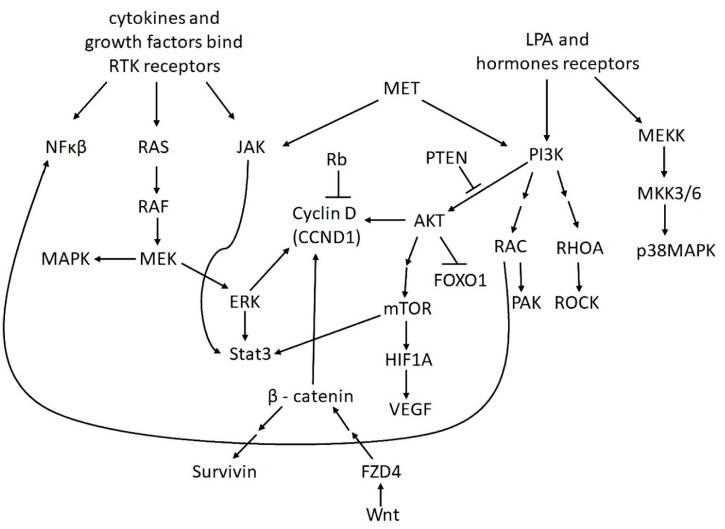
Signaling pathways involved in ovarian cancer pathogenesis. Straight arrows indicate activation of gene (protein) expression and blunt arrows indicate inhibition of gene (protein) expression. Two consecutive arrows mean that there may be other participants in the process, i.e. the connection is not direct, but through intermediaries.

The most important signaling pathways in OvCa and some of their connections are shown in Figure 1.

## 4. Mechanisms and Pathways Associated with OvCa Metastasis

A feature of metastasis in OvCa is the absence of a barrier between the primary tumor and the abdominal cavity. Multicellular aggregates, enriched with cells with stem properties, leave the primary tumor and can be implanted into the peritoneal membrane. EMT plays an important role in metastasis and involves the transition from an immobile polarized epithelial cell associated with the environment to a mobile cell with mesenchymal morphology. At the same time, the cells enhance their invasion ability and acquire stem properties and resistance to chemotherapy. There is a decrease in the expression of proteins such as E-cadherin and γ-catenin, which are responsible for intercellular contacts, and an increase in the expression of proteins such as vimentin, N-cadherin, and fibronectin and in the activity of some metalloproteinases of extracellular matrix (ECM). The mechanism of metastasis in OvCa was discussed in more detail earlier, in our previous review work [32].

EMT is stimulated by various growth factors, such as TGF-β, PDGF, and FGFR, and by NF-κB, WNT, NOTCH, and Hh proteins. It can be reversed by suppressing components of the Wnt and Notch signaling pathways [44,45]. For the role of the Wnt pathway in the regulation of stem cells in EMT, see also Reference [46].

**Figure 2:**
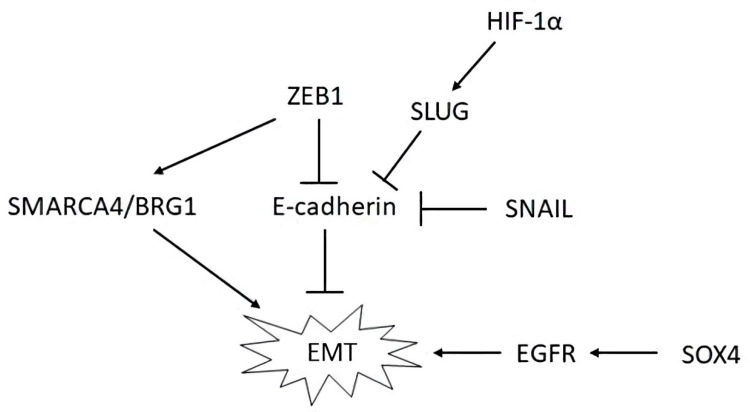
Signaling pathways involved in epithelial-mesenchymal transition (EMT) in ovarian cancer. Straight arrows indicate activation of gene (protein) or process and blunt arrows indicate inhibition of gene (protein) or process.

E-cadherin is responsible for calcium-dependent intercellular interactions and for maintaining the organization of the epithelial cytoskeleton. Its direct repressors include SNAIL, SLUG/SNAIL2, ZEB1, ZEB2, and E47 [44,45]. ZEB1 represses the E-cadherin promoter and also stimulates EMT through the involvement of SMARCA4/BRG1, SOX4 regulates, in particular, the epidermal growth factor receptor (EGFR), while HIF-1α regulates SLUG, among others, through proteasome-mediated degradation [44,45]. Some interactions affecting EMT in OvCa are shown in Figure 2.

HMGA2, acting as a transcription factor, also induces EMT [47]. Activation of the PI3K/AKT/mTOR pathway was also associated with EMT and the presence of stem properties in cells, in particular greater chemoresistance. Inhibition of this pathway reverses EMT and increases sensitivity to chemotherapy [48]. STAT3 activation due to the action of proinflammatory cytokines, growth factors, and oxidative stress stimulates important OvCa metastasis processes such as EMT, degradation of extracellular matrix, and the acquisition of stem traits [49].

The specificity of metastasis in OvCa is that EMT often does not reach the end, and both mesenchymal and epithelial determinants are often present in cells. In addition, in metastases in the abdominal cavity and peritoneum, EMT can partially revert by mesenchymal-epithelial transition [44,45].

A change in the migration and invasive activity of OvCa cells is significant for metastasis and is also closely related to EMT. As already mentioned, growth factors can stimulate this transition. For example, the growth factor BDNF, when overexpressed, activates the small GTPase Rac1, leading to the reorganization of the actin cytoskeleton due to the AKT/mTOR signaling pathway and to an increase in the invasive activity of cells [50]. Simultaneous inhibition of MEK1/2 and Src can eliminate EMT induced by active Rac1 [51].

With OvCa metastasis and the formation of ascites, aggregates of cancer cells are formed that can survive without a substrate in the event of their accumulation. The formation of spheroids serves as an analog of such aggregations under cell culture conditions. It has been repeatedly shown that spheroids are better models for testing tumor response to drugs than cell cultures on planar substrates. YAP1 is a transcriptional regulator involved in the Hippo pathway (a key component, Hippo protein kinase, controls organ size by regulating cell division and apoptosis) and is associated with spheroid formation [52].

VEGF, in addition to influencing tumor angiogenesis, also plays an important role in metastasis in OvCa. VEGF is found in OvCa tissues, omentum metastases, cystic fluid, ascites, and serum. VEGF is expressed in all malignant tumors, but not in the normal ovarian cortex or benign tumor tissues, and in OvCa, negatively correlates with survival [53]. VEGF levels in patients with ascites exceeded control by 200 times.

Cytokines have a significant impact on the creation of a favorable environment for metastases. Thus, inhibition of the expression of the cytokine CXCL12 reduces the migration and invasion of OvCa cells [54]. This cytokine is absent in normal tissue but is present in both tumor and ascites, and its expression negatively correlates with the survival rate of OvCa. CXCL12 enhances the interaction of OvCa cells with ECM components and with mesothelial cells, enabling the formation of peritoneal metastases [54].

## 5. Mechanisms and Pathways Associated with Drug Resistance in OvCa

The drugs often used in OvCa treatment are platinum salts, such as cisplatin or carboplatin, and taxanes such as paclitaxel. The first group of drugs causes DNA damage, while the second binds to β-tubulin, leading to the arrest of the cell cycle. Besides, Anti-VEGF therapy is used, and Olaparib treatment (PARPi) is applied as targeted therapy against *BRCA1/2*-mutated tumors [22], but almost all studies on resistance mechanisms concern either resistance to platinum salts and taxanes or mechanisms of general importance. The already mentioned development of resistance to therapy is largely associated with the processes occurring during EMT, in particular with the acquisition of stem properties by cells. The acquisition of stem properties leads to the expression of tubulin forms atypical for normal tissue, increased activity of ATP-binding cassette (ABC) transporters that pump out drugs from the cell, a higher efficiency of DNA repair, and a lower apoptotic activity [55,56]. Thus, the expression of SNAIL and SLUG makes cells more resistant to p53-mediated apoptosis. SNAIL expression stimulates the PI3K/AKT pathway and also inhibits apoptosis by increasing Bcl-XL expression [56].

## 6. LncRNAs in the Development and Progression of OvCa via ceRNA Model

Earlier, other authors [57,58,59] and us [32] have discussed the possible role of miRNAs in the processes involved in the development and progression of OvCa. MiRNAs participate in many biological processes including proliferation, apoptosis, EMT, metastasis, cancer stem cell, and chemoresistance. MiRNAs substantially contributed to our understanding of OvCa pathogenesis and metastasis. The inhibitory effect of miRNAs on the translation of target gene mRNAs through their direct binding in RNA-induced silencing complexes (RISCs) was revealed for multiple miRNA–mRNA pairs involved in the main signaling pathways of OvCa [7,32]. Given their resistance to the degradation by ribonucleases and availability in plasma exosomes, miRNAs may serve as emerging biomarkers for cancer detection, therapeutic assessment, and prognostic prediction [60]. Being a messenger, exosomal miRNAs are crucial for the crosstalk between cancer cells and stromal cells in tumor microenvironment [60]. However, miRNAs themselves are also subject to regulation in OvCa through various mechanisms at epigenetic, transcriptional, and post-transcriptional levels, including aberrant methylation of miRNA genes [61] and the interactions between lncRNA and miRNA [62].

Based on recent discoveries, the involvement of lncRNAs in the proliferation, apoptosis, cell cycle, migration, invasion, metastasis, and drug resistance of OvCa was established [63]. It is assumed that lncRNA can perform a variety of functions in OvCa through different mechanisms, including interactions with DNA, proteins, and RNA [64]. However, increasing experimental data are accumulating, indicating the applicability and significance of the ceRNA model in OvCa [20,65]. According to this model, lncRNA is involved in the regulation of the expression of protein-coding genes through direct binding along the lncRNA/miRNA/mRNA (target protein) axis, that is, with the direct participation of miRNA [20,65,66]. In this case, lncRNA can compete with the mRNA of the target protein gene for binding to miRNA, in which common miRNA-response elements can participate. According to the ceRNA model, direct binding of miRNAs with both lncRNA and mRNA is assumed. The prognostic value of the regulatory ceRNA network in OvCa was noted, including the determination of chemosensitivity [66,67].

To prove these direct connections, as a rule, a set of methods is used (see for example Reference [68]). Thus, the levels of lncRNA, miRNA, and mRNA in vivo and in vitro are usually estimated by quantitative reverse transcription polymerase chain reaction (qRT-PCR) or using microarrays. Western blot analysis is used to determine proteins. Bioinformatics methods are used to predict possible interactions between RNAs of different types, while correlation analysis of lncRNA, miRNA, and mRNA levels and gain- or loss-of-function assays and dual-luciferase reporter tests are used to experimentally confirm “potential interactions”. In addition, the RNA pull-down method was developed specifically to detect RNAs that bind to lncRNA. Due to the fact that interactions between different ncRNAs occur in a complex with proteins of the Argonaute family, methods that confirm direct RNA–protein interactions are in demand, in particular, the analysis with the RIP assay. For example, the direct binding of miR-525-5p to MAGI2-AS3 and negative regulation of miR-525-5p by MAGI2-AS3 in human ovarian cancer cell lines was proven using transfection by plasmids, qRT-PCR, dual-luciferase reporter assay, pull-down assay, and RIP with anti-AGO2 vs anti-IgG antibodies [69]. Thus, luciferase activity in OvCa cells transfected with MAGI2-AS3-WT and miR-525-5p mimics was decreased, while no evident changes were seen in cells transfected with MAGI2-AS3-Mut and miR-525-5p mimics. Results of pull-down assays revealed that biotinylated miR-525-5p-WT probe, unlike biotinylated miR-525-5p-Mut probe, could precipitate MAGI2-AS3. RIP data showed that MAGI2-AS3 and miR-525-5p enriched in AGO2 protein but not in IgG [69].

When simulating the effect of a particular RNA overexpression, both in vitro and in vivo (on xenografts), as a rule, mimetics or genetically engineered constructs are used. The reduced expression of the target gene is achieved by knocking it down with the help of the corresponding siRNAs or hairpin-forming short RNAs (shRNAs). Evidence of the biological significance of a particular interaction is considered to be the “cancellation” of the effect of lncRNA overexpression (in whole or in part) upon overexpression of the target miRNA and the effect of suppressing the lncRNA expression (in whole or in part) upon a decrease in the expression of the miRNA to which it binds.

In the initial phases, bioinformatics methods can be used to search for potentially interacting RNAs, but the standard of modern research requires the subsequent confirmation of this interaction using a combination of the above-mentioned biochemical methods.

Here, we have summarized the data of those studies in which the direct binding of lncRNA and miRNA was confirmed; mainly, we considered as mRNA targets those for which direct binding to miRNA was validated using the already mentioned methods: dual-luciferase reporter assay, RNA pull-down, and RIP assay (see for example References [70,71]).

## 7. Suppressor lncRNAs as ceRNAs in Ovarian Cancer

LncRNA, similarly to miRNA and genes encoding proteins, can exhibit a tumor-promoting oncogenic or a tumor-suppressing effect in OvCa cells. Table 1 shows the lncRNA/miRNA/mRNA axes identified for the group of oncosuppressive lncRNAs in OvCa. For each suppressor lncRNA, reduced expression in cell lines and/or clinical tumor specimens and an inhibitory effect on proliferation are shown. The vast majority of suppressor lncRNAs are involved in suppressing OvCa progression, migration, invasion, EMT, and metastasis. Suppressor lncRNAs, acting according to the ceRNA model, suppress the expression and functional activity of oncogenic miRNA, with which they directly bind, which further leads to an increase in the mRNA level of the target cancer suppressor gene and the protein encoded by it.

Thus, lncRNA ADAMTS9-AS2 (ADAM metallopeptidase with thrombospondin type 1 motif 9 (ADAMTS9) antisense RNA 2) showed reduced expression in OvCa cell lines relative to the human ovarian surface epithelial cell line (IOSE80). ADAMTS9-AS2 expression was also significantly reduced in OvCa tissues according to qRT-PCR data [72]. A decrease in the lncRNA ADAMTS9-AS2 level in clinical OvCa specimens correlated with advanced FIGO (International Federation of Gynecology and Obstetrics) stage, lymph node metastases, and poor overall survival. In SKOV3 and OVCAR3 cell lines, it was shown that ADAMTS9-AS2 overexpression suppresses proliferation, invasion, and EMT, inducing E-cadherin expression and decreasing Vimentin expression, both in mRNA and protein levels. Moreover, an over-expressing ADAMTS9-AS2 cell line reduced tumor growth in vivo in BALB/c nude mice [72]. All these data indicated the oncosuppressive properties of ADAMTS9-AS2 and involvement in the inhibition of OvCa progression and metastasis (Table 1).

Furthermore, the authors determined a mechanism by which this lncRNA can suppress OvCa and its progression, through the axis lncRNA ADAMTS9-AS2/miR-182-5p/FOXF2 (forkhead-related transcription factor 2). In addition to analyzing the mutual influence of the components of this axis, direct binding of ADAMTS9-AS2 to miR-182-5p was validated using a set of experiments (dual-luciferase reporter, RIP, and RNA pull-down assays), and *FOXF2* mRNA as a direct target of miR-182-5p was confirmed using luciferase reporter assay [72].

LncRNA EPB41L4A-AS2 (erythrocyte membrane protein band 4.1 like 4A (EPB41L4A) antisense RNA 2) was expressed at a low level in OvCa tissues and cells [73]. SKOV3 cells overexpressing EPB41L4A-AS2 showed reduced proliferation, colony formation, and reduced tumor formation in vivo, which was observed using tumor xenograft in nude mice. Besides, overexpressed EPB41L4A-AS2 inhibited the proliferation, migration, and invasion of SKOV3 OvCa cells and, therefore, could suppress the progression and metastasis of OvCa. Direct interaction forming the axis EPB41L4A-AS2/miR-103a/RUNX1T1 (runt-related transcription factor 1 (RUNX1) partner transcriptional co-repressor 1) was proven using combined methods, including RIP and dual-luciferase reporter gene assays [73]. Thus, overexpressed EPB41L4A-AS2 prevents the progression of OvCa by activating RUNX1T1 via miR-103a (Table 1).

**Figure 3:**
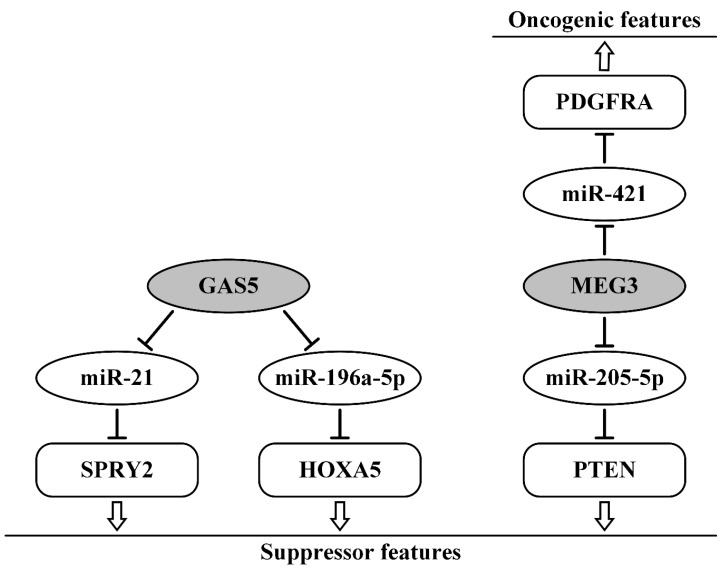
Two axes, regulated by lncRNA GAS5 (growth arrest-specific 5), both with suppressive functions (left), and two lncRNA MEG3 (maternally expressed 3) axes with oncogenic and suppressive functions (right). Blunt arrows indicate inhibition of miRNA or gene (protein) expression and straight double arrows indicate manifestation by a given gene of suppressive or oncogenic properties.

For lncRNA GAS5 (growth arrest-specific transcript 5), a tumor-suppressing function and a role in the inhibition of OvCa progression have also been shown [74,75]. Moreover, two regulatory axes were found GAS5/miR-21/SPRY2 (sprouty homolog 2) and GAS5/miR-196a-5p/HOXA5 (homeobox A5), through which GAS5 can perform its functions (Table 1, Figure 3, left). Decreased GAS5 expression was associated with larger tumor size (≥5 cm), advanced clinical stage (III-IV), and poor prognosis of OvCa patients. GAS5 suppressive effects on cell proliferation, viability, as well as migration, invasion, and EMT were established by functional studies in vitro using OvCa-derived A2780 cells and in vivo with animal models. Direct interactions in both axes were validated using expression-modulating studies and the dual-luciferase reporter gene assay as well [74,75].

Overexpression of lncRNA HAND2-AS1 (heart and neural crest derivatives expressed 2 (HAND2) antisense RNA 1) enhances apoptosis, blocks proliferation, and inhibits cell migration and invasion of OvCa-derived ES-2 cells and SKOV3 cell line in vitro [68]. The tumor xenograft was also used in nude mice to show that HAND2-AS1 upregulation suppressed tumorigenesis in vivo. The interactions in axis HAND2-AS1/miR-340-5p/BCL2L11 in OvCa (Table 1) were shown using gain- or loss-of-function assays. RIP assay showed that HAND2-AS1 could bind to both AGO2 protein and miR-340. The dual-luciferase reporter gene and RNA pull-down assays were used to verify whether the targeting relationship between miR-340-5p and HAND2-AS1 and between miR-340-5p and BCL2L11 mRNA existed [68]. From the predicted data in online software, a putative binding region was found between the miR-340-5p and the *BCL2L11* 3′-untranslated region (UTR) and HAND2-AS1 as well. As a whole, this data suggests HAND2-AS1 as a suppressive lncRNA in OvCa via upregulation of BCL2L11, through competitively binding to miR-340-5p [68]. Interestingly, the suppressor function of HAND2-AS1 is suppressed by hypermethylation of the gene of this lncRNA, which reduces its expression in tissues and OvCa cell line SKOV3 [76].

LncRNA HOTAIRM1 (HOXA transcript antisense RNA, myeloid-specific 1), under-expressed in OvCa cells and tumor tissues, also belongs to suppressor lncRNA (Table 1). Decreased HOTAIRM1 expression was shown to be associated with lymphatic metastasis and advanced OvCa FIGO stages [77]. Overexpression of HOTAIRM1 promoted apoptosis and suppressed proliferation, invasion, and metastasis in vitro and HOTAIRM1 suppressed tumor growth in vivo. Luciferase reporter, RIP, and RNA pull-down assays were used to confirm the interactions of miR-106a-5p with HOTAIRM1 and with ARHGAP24 (Rho GTPase activating protein 24) in the axis HOTAIRM1/miR-106a-5p/ARHGAP24 [77].

The long intergenic non-coding RNA LINC01088 was shown to be downregulated in ovarian tumor tissues (Table 1). Upregulated LINC01088 decreased proliferation in vitro and inhibited the growth of OvCa xenografts in nude mice. LINC01088 might act as a tumor suppressor in OvCa [78]. The LINC01088/miR-24-1-5p/PAK4 (p21-activated kinase 4) axis was validated using Western blotting and the luciferase reporter assay [78].

The long intergenic LINC01125 was under-expressed, especially in cisplatin (CDDP)-resistant OvCa cell lines and tumor tissues [79]. Overexpression of LINC01125 inhibited OvCa cell proliferation and enhanced cisplatin/Taxol sensitivity (Table 1). LINC01125 promoted apoptosis, might act as a tumor suppressor, and enhanced the cisplatin sensitivity of OvCa cells. The LINC01125/miR-1972 axis was found by bioinformatics analysis and qRT-PCR and validated by dual-luciferase reporter and RIP assays [79].

The long intergenic LINC01133 is scarcely expressed in OvCa tissues and cells. LINC01133 repressed cell proliferation, colony formation, invasion, migration, and tumorigenic ability in vitro and tumor formation and metastasis in vivo on tumor xenograft in nude mice (Table 1). The LINC01133/miR-205/LRRK2 (leucine rich repeat kinase 2) axis was proven via gain-of-function and loss-of-function experiments, Western blotting, and dual-luciferase reporter, RNA pull-down, and RIP assays [80]. LINC01133 suppressed OvCa development and progression through this axis, inhibiting miR-205 and elevating the mRNA of LRRK2, which is the miR-205 target gene.

LncRNA MAGI2-AS3 (membrane-associated guanylate kinase, WW and PDZ domain-containing 2 (MAGI2) antisense RNA 3) has been shown to be downregulated by hypermethylation, reduces migration of OvCa cell lines, and has been recognized as suppressive [81]. This lncRNA has also been shown to reduce the ability of the transfected cells to adhere to extracellular matrix mimicked by fibronectin or collagen. The three miRNAs, miR-15b-5p, miR-374a-5p, and miR-374b-5p, have been shown to interact directly or indirectly with MAGI2-AS3 based on correlation analysis of lncRNA and miRNA levels and gain- or loss-of-function assays [81]. Potential target genes such as *PTEN*, *HOXA5*, and *RECK* were selected bioinformatically and based on data on other types of cancer. It is assumed that MAGI2-AS3 operates in the ceRNA model, although direct binding has not been sufficiently validated (Table 1). Another study also confirmed the suppressive function of lncRNA MAGI2-AS3 and revealed its ability to inhibit invasion and induce an arrested cell cycle transition from G0/G1 to S or G2/M phase [69]. Using a set of methods (transfection by plasmids, qRT-PCR, dual-luciferase reporter assay, pull-down, and RIP assay with anti-AGO2 vs anti-IgG antibodies), direct binding of MAGI2-AS3 to miR-525-5p was shown, which reduces the level of this miRNA [69]. Moreover, miR-525-5p protein-target MXD1 (MYC-associated factor X (MAX) dimerization protein 1) was identified because its mRNA and protein levels were downregulated by miR-525-5p and positively correlated with MAGI2-AS3 in OvCa cells, which was shown using qRT-PCR, transfection, Western blotting, and luciferase reporter, RNA pull-down, and RIP assays [69]. For example, RIP assays demonstrated that MAGI2-AS3, miR-525-5p, and MXD1 coexisted in RNA-induced silencing complexes (RISCs). In addition, it was found that MAGI2-AS3 could repress MYC transcriptional activity by strengthening the interaction of MXD1 with MAX. Therefore, MAGI2-AS3 can suppress OvCa progression involving two axes [69,81], including the most validated MAGI2-AS3/miR-525-5p/MXD1 that also can contribute to MYC signaling [69].

The long intergenic non-coding RNA MIR503HG (miR-503 host gene) was shown to be downregulated in OvCa tissues. Patients with the lowest level of this lncRNA were characterized by poor survival [82] (Table 1). In addition, MIR503HG suppressed OvCa cell invasion and migration. The regulatory interaction between MIR503HG and miR-31-5p was predicted by IntaRNA (http://rna.informatik.uni-freiburg.de/IntaRNA/Input.jsp). Indeed, the authors observed that MIR503HG may form strong base-pairing with miR-31-5p and that expression of MIR503HG negatively correlated with miR-31-5p expression. Direct interaction between MIR503HG and miR-31-5p was validated by gain- or loss-of-function assays and the dual-luciferase assay. Interestingly, methylation-specific PCR showed that overexpression of MIR503HG led to an increased methylation level of the miR-31-5p gene. Therefore, MIR503HG may inhibit miR-31-5p in OvCa in two different ways: by sponging mature miR-31-5p and promoting the methylation of the miR-31-5p gene [82] (Table 1).

The lncRNA MORT (mortal obligate RNA transcript) decreased expression in OvCa tumors, showed suppressive function, as well as binding to oncogenic miR-21, which was shown by the inverse correlation of the expression of MORT and miR-21 in tissues of 70 patients and through the gain- or loss-of-function assays in cell transfection experiments [83] (Table 1).

The lncRNA WDFY3-AS2 (WD repeat and FYVE domain-containing 3 (WDFY3) antisense RNA 2) was less expressed in OvCa tissues than in normal tissues and was lower in the four OvCa cell lines, A2780, CP70, SKOV3, and CAOV3, compared with the normal ovarian epithelial cell line IOSE80 [84]. It was established that lncRNA WDFY3-AS2 overexpression inhibits proliferation, migration, invasion, and EMT of OvCa cells in vitro, and also inhibits the tumorigenic ability of OvCa cells in vivo, which demonstrates suppressing and anti-metastatic features of WDFY3-AS2 (Table 1). The lncRNA WDFY3-AS2/miR-18a/RORA (RAR-related orphan receptor A, also known as RORα, or NR1F1) axis was shown and confirmed using complex methods including luciferase reporter, RNA pull-down, and RIP assays [84]. Both WDFY3-AS2 and RORA are involved in inhibiting the progression of OvCa and represent ceRNAs, competing for binding of oncogenic miR-18a.

The lncRNA XIST (X inactive specific transcript) showed very low expression in OvCa cell lines and tissues according to Reference [85]. XIST overexpression inhibited OvCa proliferation, migration, and invasion, suppressed OvCa xenograft and metastasis in vivo, and also increased OvCa chemosensitivity. Using previously reported data about *PTEN* (phosphatase and tensin homolog) as a direct target of miR-214-3p [86] and data about direct binding XIST with miR-214-3p, which was validated via gain- or loss-of-function assays and the luciferase reporter gene assay [85], we can suggest that XIST is involved in the anticancer effect in OvCa cells through the axis XIST/miR-214-3p/PTEN (Table 1).

The lncRNA MEG3 (maternally expressed 3) was shown to be highly expressed in OvCa cells [87]. In this paper, the MEG3/miR-421/PDGFRA (platelet-derived growth factor receptor α) axis was validated via luciferase reporter assays (Table 1, Figure 3, right). The authors suggest that MEG3 induces tumor cell proliferation, invasion, metastasis, and angiogenesis via this axis. They proposed anisomycin as an inhibitor of the effect of MEG3 and PDGFRA on angiogenesis and cancer progression [87]. On the contrary, reduced expression of MEG3 in OvCa cell lines and tissues and the ability of MEG3 to decrease migration and invasion were shown recently [88]. The suppressor function of MEG3, realized by direct interaction with oncogenic miR-205-5p, was validated via a luciferase reporter assay [88]. Using data about suppressor PTEN as a direct target of miR-205-5p [89] and recent data from Reference [88], we can suggest that MEG3 is involved in the inhibition of cell viability, migration, and invasion, and in the promotion of apoptosis in OvCa cells through the axis MEG3/miR-205-5p/PTEN (Figure 3, right).

The contradictions in the properties of MEG3 according to the data of different works can be associated with the dual behavior of this lncRNA or with the difference in experimental approaches. Data on hypermethylation of MEG3 in OvCa [91] support its suppressor function.

It should be noted that almost all of the detected suppressor lncRNAs acting in the ceRNA model have an inhibitory effect on the progression and metastasis of OvCa. In addition, some of them (GAS5, LINC01125, MEG3, and XIST) can be used as predictors of survival and/or response to therapy in OvCa patients.

## 8. Oncogenic lncRNAs as ceRNAs in Ovarian Cancer

For the group of oncogenic lncRNAs, the lncRNA/miRNA/mRNA axes are given, in which the direct binding of miRNA to lncRNA and to mRNA proteins (if it is given) was confirmed quite convincingly with several methods, as in gain- or loss-of-function, dual-luciferase reporter, RIP, and RNA pull-down assays (Table 2).

As can be seen from Table 2, each pro-oncogenic lncRNA shows increased expression in cell lines and tumors and a positive effect on proliferation. The overwhelming majority of lncRNAs are involved in the progression of OvCa, in increased migration, invasion, EMT, and metastasis in OvCa patients, which has been shown in many examples both in vitro and in vivo. A number of lncRNAs have been studied in several works and two or more lncRNA/miRNA/mRNA axes have been determined each (Table 2). We used them as examples for a more detailed consideration of the properties of oncogenic lncRNAs and their involvement in the pathogenesis and progression of OvCa.

**Figure 4:**
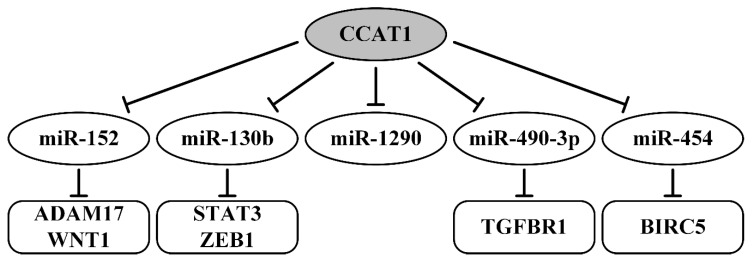
Set of multiple axes regulated by the oncogenic lncRNA CCAT1 (colon cancer-associated transcript 1) in ovarian cancer. Blunt arrows indicate inhibition of miRNA or gene (protein) expression.

The lncRNA CCAT1 (colon cancer-associated transcript 1) as a typical proto-oncogene has been reported to be highly expressed in OvCa tissues and cell lines (Table 1). CCAT1 was correlated with FIGO stage, histological grade, lymph node metastasis, and shorter survival, and was defined as an independent prognostic factor [95]. CCAT1 upregulation promoted cell migration and invasion as well as reduced E-cadherin expression (epithelial marker) and promoted vimentin and N-cadherin expression (mesenchymal markers) [95]. Using gain- or loss-of-function assays, Western blotting, and luciferase reporter assays, miR-152 and miR-130b have been confirmed as direct targets of CCAT1 [95]. In addition, in the same way, it was shown that miR-152 targets ADAM17 (A disintegrin and metalloproteinase 17) and WNT1 (proto-oncogene, Wnt family member 1) and that miR-130b targets transcription factors STAT1 (signal transducer and activator of transcription 1) and ZEB1 (zinc finger E-box binding homeobox 1). MiRNA response-elements of miR-152 and miR-130b have been identified in the 3’-UTR of these genes respectively, and in CCAT1 as well [95]. Thus, in this work, two-branched axes were identified: CCAT1/miR-152/ADAM17 (WNT1) and CCAT1/miR-130b/STAT3 (ZEB1), which are also shown in Figure 4.

Three more studies confirmed the involvement of lncRNA CCAT1 in EMT, migration, invasion, metastasis, association with advanced FIGO stages, and poor survival [96,97,98]. Two novel axes were identified and validated via complex methods, including the luciferase reporter assay. This confirmed the direct interaction of CCAT1 or TGF-β1 with miR-490-3p [97] as well as the direct interactions among CCAT1, miR-454, and BIRC5 [98]. Inhibiting binding CCAT1 with miR-1290 was shown using plasmid transfection and gain- or loss-of-function assays [96]. In a recent xenograft work, the suppressing effect of CCAT1 on chemoresistance to cisplatin was shown in vitro and in vivo [98]. Therefore, lncRNA CCAT1 is involved in the regulation of a number of genes through five regulator axes, which are reported in Figure 4.

The lncRNA CDKN2B-AS1 (cyclin-dependent kinase inhibitor 2B antisense RNA 1) could directly interact with miR-411-3p, which was confirmed through an inverse correlation between miR-411-3p and CDKN2B-AS1 level, gain- or loss-of-function, and luciferase reporter assays [100]. Moreover, CDKN2B-AS1 contain miRNA-response elements complementary to miR-411-3p regions. In addition, CDKN2B-AS1 enhanced migration, invasion, metastasis OvCa cells, and association of CDKN2B-AS1 with the tumor growth was demonstrated by in vivo experiments [100]. The effect of CDKN2B-AS1 and miR-411-3p on the HIF-1α/VEGF/P38 axis was indicated. The direct interaction of miR-143-3p with both CDKN2B-AS1 and SMAD3 was demonstrated by bioinformatics, qRT-PCR, and Western blotting analyses, gain- and loss-of-function, and luciferase reporter assays [101]. CDKN2B-AS1 was upregulated in OvCa and correlated with clinicopathologic features [101]. CDKN2B-AS1 promoted tumor growth, invasion, and migration by regulation of the miR-143-3p/SMAD3 axis and is considered a predictor of poor prognosis. Thus, the two axes CDKN2B-AS1/miR-411-3p, also activating HIF-1α/VEGF/P38 signaling, and CDKN2B-AS1/miR-143-3p/SMAD3 were involved in OvCa progression (Table 2).

The imprinted lncRNA H19 is highly expressed in OvCa tissues and cell lines, promotes cell migration and invasiveness, and functions as an oncogene. Two axes were determined through which H19 contributes to proliferation and progression of OvCa as ceRNA: H19/miR-370-3p and H19/miR-324-5p/PKM2 (Pyruvate kinase isozyme M2) [109,110] (Table 2). Either H19 overexpression or miR-370-3p knockdown promoted TGF-β-induced EMT [109]. Thus, it was shown that the H19 knockdown increased the level of epithelial marker E-cadherin and decreased the levels of mesenchymal markers SNAIL and vimentin in TGF-β1-stimulated SKOV3 and OVCAR3 cells. Of interest, at least two miRNA response elements were detected in the 3’-UTR of H19 to predict duplex formation between the wild-type H19 and miR-370-3p. Direct binding of H19 with miR-370-3p was confirmed via luciferase reporter assay [109]. Besides, H19 contributes to aerobic glycolysis (Warburg effect) and OvCa progression by increasing PKM2 mediated by miR-324-5p, and this was proven in vitro and in vivo using the xenograft tumor model [110]. In addition, it was shown that ginsenoside 20(S)-Rg3 inhibited the Warburg effect via the axis H19/miR-324-5p/PKM2 to suppress OvCa growth. Direct binding of miR-324-5p to H19 and PKM2 was validated using dual-luciferase reporter and RIP assays [110].

**Figure 5:**
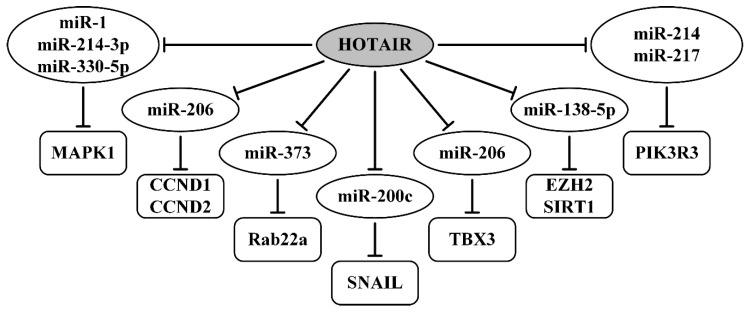
Set of multiple axes regulated by the oncogenic lncRNA HOTAIR (HOX transcript antisense intergenic RNA) in ovarian cancer. Blunt arrows indicate inhibition of miRNA or gene (protein) expression.

The lncRNA HOTAIR (HOX transcript antisense intergenic RNA) is encoded by a gene located within the homeobox C (HOXC) gene cluster on Chromosome 12 and is 2.2 kb long. HOTAIR was shown as upregulated in OvCa cell lines, and its silencing inhibited the proliferation, migration, and invasion in SKOV3 cells [114]. Specific crosstalk between lncRNA HOTAIR and mRNA of MAPK1 (mitogen-activated protein kinase 1) through competition for miR-1, miR-214-3p, and miR-330-5p binding was established using bioinformatics, qRT-PCR, Western blotting analyses, gain- or loss-of-function, and luciferase reporter assays. It was demonstrated that silencing HOTAIR or MAPK1 increased the expression of miR-1, miR-214-3p, or miR-330-5p, and also inhibited proliferation, migration, and invasion in SKOV3 cells [114]. The axes of direct binding between lncRNA HOTAIR, miRNAs (miR-1, miR-214-3p, miR-330-5p), and *MAPK1* mRNA are represented in Table 2 and Figure 5. Further studies confirmed the oncogenic features of HOTAIR and revealed new axes through which this lncRNA promotes OvCa cells’ proliferation, migration, and invasion ability. Novel axes HOTAIR/miR-214(miR-217)/PIK3R3 (phosphoinositide-3-kinase regulatory subunit 3) were also validated using complex methods, including luciferase reporter assays [115]. These first two works showed HOTAIR as a ceRNA [114,115], isolated several miRNAs with which it directly binds, and identified the target protein (MAPK1 or PIK3R3) as the point of intersection of these axes.

In another study [116], the effect of HOTAIR on the progression and metastasis of OvCa cells was examined by overexpression, knockdown, or small interfering RNA interference experiments. A novel axis HOTAIR/miR-373/Rab22a (Ras-related protein of small GTPases of the RAB family) and ceRNAs mechanism involvement were validated with the use of the luciferase reporter assay [116]. Another axis, HOTAIR/miR-206/CCND1 (CCND2) (cyclin D1 and D2), involved in the regulatory functions of HOTAIR in the progression and metastasis of OvCa, has been determined using a range of methods, including bioinformatics analysis, qRT-PCR, northern blotting, Western blotting, expression level modulation, and the dual-luciferase reporter gene assay [117].

The axis HOTAIR/miR-200c/SNAIL (or SNAI1, zinc finger transcription factor), involved in OvCa invasion, was demonstrated using transduced lentivirus-miR-200c, gain- or loss-of-function assays, EMT markers, and tumorigenicity of SKOV3 cells in xenograft tumor studies [118]. In recent work, it was shown that miR-138-5p could directly bind to HOTAIR and 3’-UTR of EZH2 (enhancer of zeste homolog 2, a histone-lysine N-methyltransferase) and SIRT1 (NAD-dependent deacetylase sirtuin 1), which was validated by the dual-luciferase reporter assay [119]. Moreover, using cisplatin-resistant OvCa cell lines, it was shown that HOTAIR reduces cisplatin chemosensitivity of OvCa cells via the axis HOTAIR/miR-138-5p/EZH2 (SIRT1) [119].

OvCa stem cells may contribute to metastasis and chemo-resistance [184]. HOTAIR was highly upregulated in the OvCa stem cells and its inhibition caused a decrease in sphere-formation ability, tumorigenicity in vivo, and cisplatin-resistance [120]. Aldehyde dehydrogenase activity was used to test sphere-formation ability. TBX3 (T-box transcription factor 3) was also upregulated and positively correlated with HOTAIR in the OvCa stem cells. Both HOTAIR and TBX3 maintained OvCa cell stemness through the axis HOTAIR/miR-206/TBX3 (Table 2). Direct binding in this axis was validated by advanced methods, including the dual-luciferase reporter gene assay [120]. All HOTAIR-regulating axes currently known (PubMed, August–September 2020) are shown in Figure 5.

LncRNA HOXD-AS1 (HOXD (Homeobox D cluster) antisense RNA 1) was shown to be upregulated in OvCa tissues and cell lines. Its high expression positively correlated with advanced FIGO stage, lymph node metastasis, and poor overall survival in OvCa patients [121]. LncRNA HOXD-AS1 promoted OvCa cells’ proliferation, EMT, migration, and invasion. MiR-133a-3p acted as a direct downstream target of HOXD-AS1, which was validated using expression correlation analysis and the dual-luciferase test. HOXD-AS1 inhibition significantly decreased the expression of β-catenin, cyclin D1, and c-Myc in SKOV3 cells, indicating Wnt/β-catenin signaling as a downstream mechanism of HOXD-AS1 in OvCa [121].

The involvement of HOXD-AS1 in OvCa progression, proliferation, EMT, migration, and invasion was confirmed in two other works [122,123]. The regulatory axes HOXD-AS1/miR-608/FZD4 (frizzled family receptor 4) and HOXD-AS1/miR-186-5p/PIK3R3 (phosphatidylinositol 3-kinase, regulatory subunit 3) were revealed in these studies. The axis HOXD-AS1/miR-608/FZD4 was validated through the luciferase reporter assay and RIP using magnetic beads conjugated with human anti-AGO2 antibody [122]. Besides, in this study, it was found that HOXD-AS1 can be involved in colony formation of OVCAR3 cells and that elevated expression of HOXD-AS1 can be a marker of poor prognosis for OvCa patients [122]. The axis HOXD-AS1/miR-186-5p/PIK3R3 was validated using small interfering RNA (siRNA) and miRNA inhibitor transfections, Western blotting, and the dual-luciferase reporter assay [123]. In addition, this study showed that HOXD-AS1 is an independent marker of poor overall survival (OS) and progression-free survival (PFS) of OvCa patients. These data are summarized in Table 1. Prognostic value of high expression of HOXD-AS1 was reported in all three studies.

Recently, two studies have shown that oncogenic lncRNA KCNQ1OT1 (potassium voltage-gated channel subfamily Q member 1 opposite strand/antisense transcript 1) is highly expressed in OvCa cells and tissues and that it could promote OvCa cell proliferation, migration, and invasion, but it also inhibited cell apoptosis of SKOV3 cells in vitro and promoted OvCa tumorigenicity in vivo using the ceRNA model [125,126] (Table 2). Thus, lncRNA KCNQ1OT1 is involved in the progression of OvCa through two axes, KCNQ1OT1/miR-212-3p/LCN2 (lipocalin2) and KCNQ1OT1/miR-142-5p/CAPN10 (calpain 10). Luciferase gene reporter and RNA pull-down assays were used to validate direct binding between KCNQ1OT1 and miR-212-3p. Direct interaction between miR-212-3p and LCN2 was shown using the luciferase gene reporter assay [125]. Direct binding of miR-142-5p to KCNQ1OT1 and CAPN10 was validated by the dual-luciferase reporter assay [126]. Moreover, in both sequences, KCNQ1OT1 and CAPN10 3’ UTR, segments of complementarity to the fragment at the 5’ end of miR-142-5p were identified. In addition, KCNQ1OT1 expression levels were significantly associated with the advanced clinical stage: when high, they predicted a shorter overall survival [125,126].

The role of lncRNA LUCAT1 (lung cancer-associated transcript 1) in OvCa has also been studied in more than one work and two regulatory axes have been identified, LUCAT1/miR-612/HOXA13 and LUCAT1/miR-199a-5p, involved in OvCa pathogenesis [137,138]. It was established that LUCAT1 was highly expressed in a number of human ovarian cancer cell lines (SKOV3, OVCAR3, HEY-T30, etc.) and OvCa tissues. LUCAT1 level was positively correlated with advanced staging, metastasis, and unfavorable prognosis in OvCa patients. LUCAT1 knockdown increased the apoptotic rate and suppressed the proliferation, migration, invasion, and colony formation of SKOV3 cells [137,138]. Using online tools, a complementary binding sequence to the miR-612 seed region was detected in LUCAT1 and the 3’-UTR region of HOXA13 mRNA. The axis LUCAT1/miR-612/HOXA13 was then validated using gain- or loss-of-function and luciferase reporter assays [137]. In another study, a highly conserved binding site of miR-199a-5p in the 3’-UTR of LUCAT1 was also a good predictor of the LUCAT1/miR-199a-5p axis, which was also validated via the dual-luciferase reporter gene assay [137,138].

The effect of lncRNA MALAT1 (metastasis-associated lung adenocarcinoma transcript 1) on the development and metastasis of various cancers, including OvCa, was shown in numerous studies [185]. The regulatory function of MALAT1 as ceRNA was shown in OvCa in five research articles (Table 2). The axis MALAT1/miR-506/iASPP (Inhibitor of apoptosis-stimulating protein of p53) was validated using cell transfection with the miRNA mimics and small-interfering RNAs (siRNAs), Western blotting, real-time PCR, and luciferase reporter gene assays [139]. Of interest, more than one predicted miR-506-binding site was confirmed in the MALAT1 3′-UTR and iASPP (inhibitor of ASPP protein) 3′-UTR via luciferase reporter gene assays. While iASPP has shown inhibition of apoptotic cell death after DNA damage, MALAT1 enhances inhibition of apoptosis and can stimulate DNA synthesis of OvCa cells in vitro through the axis MALAT1/miR-506/iASPP [139].

The next study showed that MALAT1 can manifest oncogenic regulatory functions via direct binding with miR-200c, which was validated using luciferase assays [140]. Overexpression of lncRNA MALAT1 enhanced viability, migration, and invasion of OvCa cell lines and was associated with metastasis and worse prognosis in patients [140]. Another regulatory axis, MALAT1/miR-143-3p/CMPK (uridine monophosphate/cytidine monophosphate kinase), was identified in OvCa [141]. Direct binding of MALAT1 to miR-143-3p was validated via complex studies including dual-luciferase reporter assays. Interaction of miR-143-3p with CMPK protein mRNA was suggested on bioinformatics data and Western blotting studies, also by treating OVCAR3 and SKOV3 cells with miR-143-3p mimics, which significantly decreased CMPK protein expression. Thus, MALAT1 negatively regulated miR-143-3p via a sponge-like function, and in turn, released the suppression of miR-143-3p to CMPK inhibition, leading to the progression of OvCa. MALAT1 was proved to increase cell viability, migration, and invasion of OvCa cell lines and to decrease OS and PFS of patients according to the Kaplan-Meier survival curve [141].

**Figure 6:**
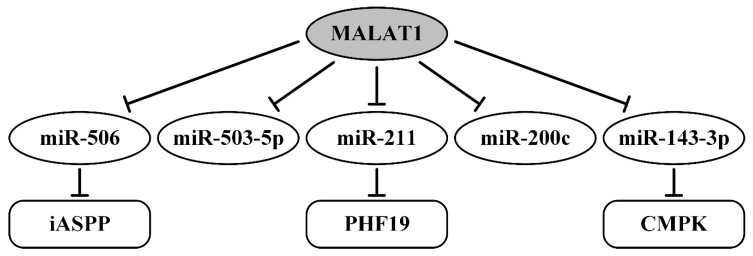
Set of multiple axes, regulated by the oncogenic lncRNA MALAT1 (metastasis-associated lung adenocarcinoma transcript 1) in ovarian cancer. Blunt arrows indicate inhibition of miRNA or gene (protein) expression.

The axis MALAT1/miR-211/PHF19 (PHD finger protein 19, a component of the polycomb group of proteins) is also involved in the progression of OvCa proliferation, migration, and xenograft growth as well. Direct binding of miR-211 with MALAT1 and with mRNA of *PHF19* was validated using the luciferase reporter assay and Western blotting analysis [142]. MALAT1 as a ceRNA can bind and inhibit miR-211 and upregulate *PHF19* expression, thus facilitating the OvCa progression. The negative correlation was also established in OvCa cells between MALAT1 and miR-503-5p level, whose direct binding was validated via luciferase and RNA pull-down assays [143]. Moreover, p-JAK and p-STAT3 were upregulated by miR-503-5p inhibitor and downregulated by si-MALAT1 (MALAT1 small-interfering RNA) in OvCa cells [143]. These data may suggest the new axis MALAT1/miR-503-5p/p-JAK (p-STAT3). MALAT1 overexpression is associated with the activation of p-JAK and p-STAT3. It means that MALAT1 can contribute to apoptosis suppression and high proliferation through the JAK2/STAT3 signaling pathway mediated by miR-503-5p [143]. All five axes regulating by MALAT1 currently known (PubMed, August–September 2020) are described in Table 2 and Figure 6.

**Figure 7:**
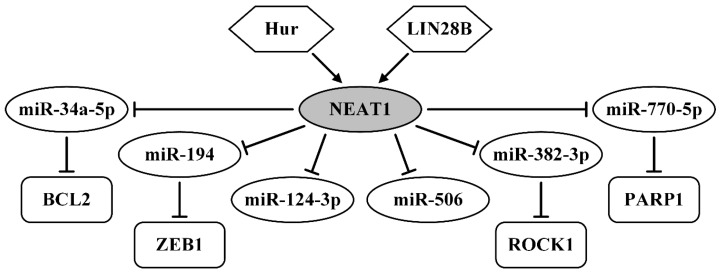
Set of multiple axes regulated by the oncogenic lncRNA NEAT1 (nuclear-enriched abundant transcript 1). RNA-binding proteins (RBP) Hur (Hu antigen R) and LIN28B (Lin-28 homolog B), stabilizing NEAT1, are also shown. Blunt arrows indicate inhibition of miRNA or gene (protein) expression and straight arrows indicate stabilizing and activating effect of RBP Hur and LIN28B.

LncRNA NEAT1 (nuclear-enriched abundant transcript 1) is an intranuclear lncRNA that participates in precursor RNA splicing. The regulatory function of NEAT1 through ceRNA was identified in six studies, which described 6 axes: Hur/NEAT1/miR-124-3p, NEAT1/miR-34a-5p/BCL2 (B-cell lymphoma-2), NEAT1/miR-194/ZEB1 (zinc finger E-box-binding homeobox 1), NEAT1/miR-382-3p/ROCK1 (rho-associated coiled-coil containing protein kinase 1), LIN28B/NEAT1/miR-506, and NEAT1/miR-770-5p/PARP1 (poly adenosine diphosphate-ribose polymerase 1), revealing interactions of NEAT with six miRNAs: miR-124-3p, miR-34a-5p, miR-194, miR-382-3p, miR-506, and miR-770-5p (Table 2). In all these studies, direct binding of each pointed miRNA with NEAT and of corresponding miRNAs with corresponding target genes (*BCL2*, *ZEB1*, *ROCK1,* and *PARP1*) was validated using qRT-PCR, transfection, a series of luciferase reporter assays, and other methods [148,149,150,151,152,153]. In these works, NEAT1 was shown to be upregulated in cell lines and tumors of OvCa patients, and its expression was associated with the FIGO stage and lymph node metastasis [148] (Table 2). A promoter effect of NEAT1 on proliferation, migration, and invasion of OvCa cells was observed in vitro [148,151,152]. Besides, NEAT1 can increase cells in the S phase and suppress apoptosis via the NEAT1/miR-34a-5p/BCL2 axis [149]. The lncRNA NEAT1 can also enhance tumor growth in vivo and can be used as a marker of poor prognosis [152]. In addition, NEAT1 increases paclitaxel resistance and cisplatin resistance through the NEAT1/miR-194/ZEB1 and NEAT1/miR-770-5p/PARP1 axes, as shown in vitro and in vivo [150,153]. It is also worth mentioning that in these works, it was found that the upregulation of NEAT1 in OvCa can be mediated by RNA-binding proteins (RBP), namely Hur (Hu antigen R) and LIN28B (Lin-28 homolog B), which bound to and stabilized NEAT1 [148,152]. All 6 axes of NEAT1 in OvCa are shown in Figure 7.

LncRNA NORAD (noncoding RNA activated by DNA damage) was upregulated in OvCa and it can increase cell proliferation, cell-cycle transition, bufalin chemoresistance, and xenograft growth [154] (Table 2). As a ceRNA, NORAD can bind and inhibit miR-155-5p. Two binding sites for miR-155-5p were detected in 3’UTR of NORAD sequence and their direct interaction was confirmed by an advanced approach, including the dual-luciferase reporter assay [154]. In addition, NORAD downregulation reversely upregulated miR-155-5p in lentiviral-transfected OVCAR3 and ES-2 cells. These data suggested oncogenic features for lncRNA NORAD, but tumor-suppressor functions for miR-155-5p. Interestingly, this miRNA is well-known to be oncogenic in various cancers (such as colon, breast, and lung) [186,187,188]. Although, in ovarian cancer, dual functions have been reported for miR-155-5p, both oncogenic [189] and suppressive [190], which is consistent with data on the negative effect of oncogenic lncRNA NORAD on miR-155-5p [154]. Besides, lncRNA NORAD increases the proliferation, EMT, and invasion of OvCa cells by inhibiting suppressive miR-199a-3p, and direct binding miR-199a-3p with NORAD 3’UTR was validated through complex methods including the dual-luciferase reporter gene assay [155] (Table 2).

LncRNA OIP5-AS1 (Opa-interacting protein 5 antisense transcript 1) was highly expressed in OvCa cell lines and clinical samples and promoted proliferation, migration, invasion, and EMT. It suppressed apoptosis in vitro and could enhance tumor growth and metastasis in vivo in nude mice [157,158] (Table 2). OIP5-AS1 functioned as a ceRNA competing with NFIB (Nuclear factor I B) and ZNF217 (Zinc finger protein 217) for binding miR-324-3p or miR-137, respectively [157,158]. An axis OIP5-AS1/miR-324-3p/NFIB was demonstrated using qRT-PCR, Western blotting, and the dual-luciferase reporter assay [157]. The OIP5-AS1/miR-137/ZNF217 axis was validated via luciferase reporter, RNA pull-down, and RIP assays [158]. OIP5-AS1 promoted OvCa tumor growth and metastasis by upregulating either ZNF217 through binding and inhibiting of suppressor miR-137 or NFIB by dint of binding of suppressor miR-324-3p [157,158].

Upregulation of lncRNA PCAT-1 (prostate cancer-associated transcript-1) in OvCa cell lines and tissue samples and its ability to inhibit apoptosis and to promote proliferation, migration, and invasion of OvCa cells was shown in two studies (Table 2). Oncogenic lncRNA PCAT-1 can bind suppressive miRNAs such as miR-129-5p and miR-124-3p, thus limiting their level in OvCa [159,160]. Moreover, direct interaction of PCAT-1 with miR-129-5p was validated using the luciferase reporter assay [159], and interaction with miR-124-3p was demonstrated via qRT-PCR, transfection, and loss/gain of function studies [160]. Some potential targets (e.g., cyclin D1, CDK6, p53, Bax, Wnt3a, β-catenin, etc.) for the PCAT-1/miR-124-3p axis in OvCa were suggested using loss/gain of function studies and Western blotting. Consequently, the authors suggested the participation of the PCAT-1/miR-124-3p axis in Wnt/β-catenin and AKT/mTOR pathways [160].

It is also interesting to dwell on three lncRNAs: PTAF (LINC00922), PTAR (AP000695.4), and PTAL (AC004988.1), whose names contain common symbols “promoting transition-associated”, which reflects their relationship with EMT (Table 2). For all these three lncRNAs, a significant positive effect on the migration, invasion, and metastasis of OvCa cells was shown. Their axes were determined, including the relative interactions with miRNAs and mRNAs of target proteins through which this effect is carried out: axis lncRNA PTAF/miR-25/SNAI2 was validated using luciferase activity assay, pull-down assay with biotin-tagged miRNA, and xenograft model of OvCa [161]. The authors constructed a lncRNA-mediated ceRNA regulatory network for the mesenchymal subtype of serous OvCa, which includes many EMT-related protein-coding genes. The inactivation of E-cadherin was considered as a hallmark of EMT. The mode of participation of the lncRNA PTAF in promoting EMT in OvCa was the activation of *SNAI2* expression through inhibition of miR-25.

The lncRNA PTAR (pro-transition associated RNA) was found upregulated in the mesenchymal subtype OvCa samples compared with the epithelial subtype samples [162]. A novel axis, PTAR (AP000695.4)/miR-101/ZEB1, was confirmed using plasmid construct and transfection, lentiviral vector construct, the luciferase reporter assay in vitro, and tumor xenografts in mice in vivo [162]. It was clearly shown that the expression of PTAR lncRNA and *ZEB1* mRNA was significantly higher in mesenchymal samples, while miR-101, on the contrary, was noticeably higher in epithelial samples. A positive correlation between PTAR and ZEB1 expression and inverse correlations of miR-101 level with PTAR and with ZEB1 expression in mesenchymal OvCa samples were established, while the involvement of the PTAR/miR-101/ZEB1 axis in EMT and metastasis of OvCa was proven in vitro and in vivo [162].

LncRNA PTAL (promoting transition-associated lncRNA) was also upregulated in mesenchymal subtype samples compared with epithelial subtype samples and was involved in OvCa progression and EMT (Table 2). It was shown that the effect of PTAL on EMT was at least partially exerted through the axis PTAL/miR-101/FN1 (fibronectin1), and was validated using plasmid construct and transfection, the luciferase reporter assay, lentiviral vector construct, and the xenograft model in nude mice [163]. It was demonstrated that PTAL positively regulated the expression of FN1 through sponging miR-101 and promoted OvCa cell metastasis by activating EMT. Of note, the same miR-101 was found in the studies described above by examining PTAR and PTAL regulatory axes. The effects of PTAF, PTAR, and PTAL on the EMT, invasion, and metastasis OvCa cells suggest that these lncRNAs could be effective targets for anti-metastasis therapies in patients with OvCa. The regulatory axes of PTAF, PTAR, and PTAL involved in EMT transition are given in Table 2.

In numerous studies, lncRNA PVT1 (plasmacytoma variant translocation 1) was upregulated in OvCa cell lines and tissues and involved in the promotion of proliferation, migration, EMT, invasion, and metastasis of OvCa cells (Table 2). It was shown that oncogenic PVT1 interacts with several tumor-suppressive miRNAs, such as miR-133a [164], miR-214 [165], miR-140 [166], and miR-543 [167]; also, inhibition of suppressor miRNAs is one of the modes to enhance the progression of OvCa. Direct binding of PVT1 to miR-133a was validated via transfections, qRT-PCR, and luciferase reporter assays [164].

A negative correlation between PVT1 and miR-214 expression detected by qRT-PCR on the representative set of more than 200 OvCa samples suggested inhibition of miR-214 by PVT1 [165]. Their interaction was also confirmed using transfection and gain/loss studies. This mechanism is apparently not so simple: the authors showed that the methyltransferase EZH2 (enhancer of zeste homolog 2) mediates the interaction of PVT1 with miR-214, which was demonstrated by RIP and ChIP (chromatin immunoprecipitation) assays with antibodies against EZH2 [165]. The authors suggested that PVT1 prompted the binding of EZH2 to the miR-214 promoter, thus inhibiting miR-214 expression. This process is more complicated than the ceRNA model and suggests interaction on the epigenomic-transcriptional level in addition to post-transcriptional RNA-RNA interactions.

Direct binding of miR-140 on PVT1 was confirmed by the luciferase reporter assay and miRNA pull-down assay [166]. PVT1 inhibited miR-140 as a miRNA sponge, while transcription of PVT1 was regulated by the transcription factor FOXO4 (Table 2). The lncRNA PVT1 and predicted target SERPINI1 (serpin peptidase inhibitor-clade I (neuroserpin)-member 1) were upregulated in OvCa cell lines and tumor tissues, unlike the downregulated tumor-suppressive miR-543 [167]. A direct interaction was validated in the PVT1/miR-543/SERPINI1 axis via the dual-luciferase reporter assay [167].

Thus, the lncRNA PVT1 promotes OvCa progression through four described axes (Table 2). The cited studies [164,165,166,167] showed that PVT1 was the most amplified gene in OvCa patients, and it was highly correlated with poor survival outcomes (progression-free and overall survivals), representing a novel perspective diagnostic and prognostic biomarker (Table 2).

LncRNA RHPN1-AS1 ((Rhophilin-1 or RHO GTPase-binding protein 1) antisense RNA 1) was shown to be highly expressed in OvCa cell lines and tissues, and involved in proliferation, migration, invasion, and metastasis (Table 2). One of the mechanisms of RHPN1-AS1 participation in OvCa progression was via the RHPN1-AS1/miR-596/LETM1 (leucine zipper/EF hand-containing transmembrane-1) axis, which was investigated through gain- and loss-of-function studies in vitro and in vivo [70]. Direct binding of miR-596 to both RHPN1-AS1 and LETM1 was validated using dual-luciferase reporter and RIP assays. RHPN1-AS1 acted as a ceRNA to bind and inhibit miR-596, consequently increasing *LETM1* expression and activating the FAK/PI3K/AKT signaling pathway (Table 2). Besides, an elevated m6A level of RHPN1-AS1 was observed in OvCa cells. It was demonstrated that the m6A modification of RHPN1-AS1 increased its transcriptional stability, and partly explained its increased expression in OvCa [70]. As shown recently [71], another way for RHPN1-AS1 to promote OvCa progression is through the inhibition of miR-1299. Direct interactions between RHPN1-AS1 and miR-1299 were validated using a number of direct methods, e.g., luciferase reporter, RIP, and pull-down assays [71]. Both studies [70,71] established that high expression of lncRNA RHPN1-AS1 indicated a low survival rate (OS and DFS) and poor prognosis.

The lncRNA TTN-AS1 (titin-antisense RNA1) can also act as ceRNA. Two axes were reported for this lncRNA in OvCa: TTN-AS1/miR-139-5p/ROCK2 (Rho-associated coiled-coil-containing protein kinase 2) and TTN-AS1/mir-15b-5p/FBXW7 (F-box/WD repeat-containing protein 7) (Table 2). Direct binding in both axes has been proven using the necessary techniques, including qRT-PCR, gain- and loss-of-function, dual-luciferase reporter assays, etc. [169,176,191]. However, lncRNA TTN-AS1 showed the properties of a typical protooncogenic lncRNA, activating migration and invasion of OvCa cells in vitro and OvCa growth in vitro and in vivo, according to Reference [169]. However, according to Reference [176], this lncRNA suppressed OvCa cell proliferation, colony formation, and promoted apoptosis (Table 2). The discrepancy in the data may have methodological reasons. Moreover, suppressor miRNAs and oncogenic protein targets (like miR-139-5p and ROCK2 in Reference [169]) are more typical for TTN-AS1. Similar features are reported for TTN-AS1 in most studies about differently located tumors (see in PubMed: cancer lncRNA TTN-AS1). It is also possible that interactions along the axis TTN-AS1/mir-15b-5p (a typical oncogenic miRNA [192])/FBXW7 (a typical suppressor, associated with degradation through ubiquitination of many oncogenic targets [193]) might change the function of TTN-AS1 lncRNA, for example, due to the reverse effect of targets on lncRNA (possibly via a feedback loop), which can also explain the difference in the results obtained in Reference [176].

For the lncRNA TUG1 (taurine upregulated gene 1), the ceRNA mode of action was established in OvCa in four works published in 2020 [177,178,179,180]. In all these studies, TUG1 was upregulated in OvCa, and this oncogenic lncRNA was associated with cell migration, invasion, and metastasis (Table 2). These studies identified three regulatory axes associated with the progression of. Direct interactions in the axis TUG1/miR-29b-3p/MDM2 (mouse double minute 2 homolog: a proto-oncogene, encoding a nuclear-localized E3 ubiquitin ligase) [177,178] were confirmed by luciferase reporter and RIP assays. TUG1/miR-186-5p/ZEB1 (zinc finger E-box-binding homeobox 1, EMT-related transcription factor) and TUG1/miR-1299/NOTCH3 (notch receptor 3) axes were also verified using luciferase reporter assays [179,180]. The authors observed that MDM2, as the primary negative regulatory factor of the p53 protein, decreases the phosphorylation level of p53. In addition, it was revealed that TUG1, by targeting miR-29b-3p, induces autophagy and consequently results in paclitaxel-resistance and poorer prognosis in OvCa patients [178]. A more detailed investigation of sphere formation and cancer stem cell properties revealed the ability of TUG1 to increase the stemness of OvCa cells, at least in part through the activation of the target ZEB1 [179]. Besides, TUG1 was found to be a potential downstream target of NOTCH3, forming a miR-1299/NOTCH3/TUG1 feedback loop in the development of OvCa. Therefore, three axes were identified and involvement of TUG1 in proliferation, migration, invasion, and metastasis was demonstrated in vitro and in vivo but, in addition, the participation of TUG1 in the increase in chemoresistance and stemness of OvCa cells and the formation of a feedback loop for the TUG1/miR-1299/NOTCH3 axis is shown in the cited works [177,178,179,180].

The expression of lncRNA UCA1 (urothelial carcinoma-associated 1) was upregulated in OvCa tissues and cells (Table 2). The oncogenic UCA1 was studied as ceRNA in two works, in which its involvement in proliferation, migration, invasion, and increasing resistance to paclitaxel in OvCa cells was shown [176,181]. Silencing of UCA1 reduced the proliferation, migration, and invasion, and enhanced the apoptosis of paclitaxel-resistant OvCa cells. The lncRNA UCA1 participates in the activation of two oncogenic target genes mediated by suppressor miRNAs and through the axes UCA1/miR-129/ABCB1 (ATP binding cassette subfamily B member 1) and UCA1/miR-654-5p/SIK2 (salt-inducible kinase 2). SIK2 is a member of the adenosine 5’-monophosphate-activated protein kinase (AMPK) sub-family, which is implicated in metabolic regulation and cancer progression [176]. Direct binding of miR-654-5p with UCA1 and SIK2 was validated by the dual-luciferase reporter assay and Western blotting analysis to assess the protein level of SIK2. Similarly, using the luciferase reporter assay and Western blotting, direct binding of miR-129 to the target ABCB1 mRNA and UCA1 lncRNA was confirmed [181]. Interestingly, 8 nucleotides in the 3’-UTR of ABCB1 and 6 nucleotides in UCA1 were complementary to the 5’-end of microRNA-129. Thus, UCA1 supports the development of paclitaxel resistance in OvCa cells, at least partly through activation of different oncogenic targets, e.g., ABCB1 and SIK2 proteins, mediated by suppressor miR-129 or miR-654-5p, respectively.

Therefore, to date, we have found, in the world literature (PubMed, August–September 2020), for the participation in the pathogenesis and progression of OvCa, 72 lncRNAs acting by the ceRNA mechanism, including only 14 with suppressor and 58 with oncogenic properties (Table 1 and Table 2). Besides, 19 lncRNAs have been revealed with multiple axes, such as GAS5, CCAT1, HOTAIR, MALAT1, NEAT1, etc. (Table 1 and Table 2, Figure 3, Figure 4, Figure 5, Figure 6 and Figure 7). Several axes show the presence of binding sites for different miRNAs on the same lncRNAs (miRNA response elements) and reveal the diverse functions of these lncRNAs in OvCa. LncRNAs MEG3 and TTN-AS1 (Table 1 and Table 2) exhibited dual features: both suppressive and oncogenic, apparently, depending on the target and the cellular context.

More than 30 lncRNAs, acting by the ceRNA mechanism, turned out to be predictors of the prognosis of survival and/or factors of response to therapy in patients with OvCa. For example, among suppressive ones such as GAS5, LINC01125, and XIST, we see improved prognosis and/or increased chemo-sensitivity (Table 1). On the contrary, among oncogenic lncRNAs, acting by the ceRNA model, e.g., CCAT1, CDKN2B-AS1, EMX2OS, GIHCG, HAGLROS, HAS2-AS1, HOTAIR, HOXD-AS1, KCNQ1OT1, and many others, we can see decreased survival and/or increased chemo-resistance (Table 2).

## 9. Examples of Alternative Mechanisms of Action of lncRNAs in OvCa

Although the most studied and, apparently, the most widespread mechanism of action of lncRNAs in OvCa according to the ceRNA model is interaction with mRNA of protein genes mediated by various miRNAs, in some other cases, their effect is associated with direct interaction with proteins or mRNA. Some lncRNAs, such as GAS5 or MALAT1, have been shown to have different mechanisms of action.

In studying mechanisms of lncRNA influence other than the interactions by the ceRNA mechanism, a wide range of methods has also been used. Thus, the interaction between lncRNA GAS5 and the transcription factor E2F4 was confirmed by RNA pull-down and RIP assays, and the interaction of this factor with the PARP1 gene promoter was confirmed by the ChIP-qPCR and luciferase reporter assays [194]. Thus, GAS5 inhibits OvCa development by the same mechanism as PARP inhibitors used in OvCa therapy. This set of methods is by now evidentiary standard. Below, we will consider in more detail some works which show the nature of the interaction of lncRNA in OvCa quite convincingly.

PANDAR lncRNA (promoter of CDKN1A antisense DNA damage-activated RNA) transcription is induced by p53. In turn, PANDAR binds to the splicing factor SFRS2, which then negatively regulates p53 and its phosphorylation. As a result, the expression of p53-activated proapoptotic genes decreases, and the resistance to chemotherapy increases. Thus, a feedback loop is observed. Cisplatin resistance increases the most because cisplatin is more likely to induce an increase in PANDAR than doxorubicin or paclitaxel [195].

Increased expression of lncRNA GHET1 (gastric carcinoma high-expressed transcript 1) correlates with large tumor size and metastases in OvCa. It interacts with VHL, blocking the degradation of HIF-1α caused by it and thus increasing the level of glycolysis in OvCa cells, as well as proliferation and colony formation [196].

LncRNA DANCR (differentiation antagonizing non-protein coding RNA) directly interacts in the cytoplasm of OvCa cells with the UPF1 protein (participates in the nonsense-mediated decay process), which leads to a decrease in the UPF1 level. The result is increased cell proliferation and migration. Increased DANCR expression in patients correlates with more advanced stages and the presence of metastases [197]. The same lncRNA also acts as an oncogene, increasing the VEGF level through the ceRNA mechanism (Table 2, [102]).

In some cases, interaction occurs with the mRNA, which acts as a direct target, and not with the protein itself. Thus, it has been shown that in OvCa lncRNA, RP11-552M11.4/lnc-WDR77 (human WD-repeat domain 77) binds to mRNA BRCA2, by suppressing the expression of which it stimulates proliferation, migration, and invasion of cancer cells and suppresses apoptosis [198]. Expression of this lncRNA correlated with more advanced stages of cancer and worse OS.

Binding to certain transcription factors and subsequently activating or, conversely, repressing the transcription of certain genes is considered a fairly common mechanism of action of various lncRNAs. A database with predictions of such interactions for different types of cancer (lncRNA Modulator Atlas in Pan-cancer (LncMAP)) has even been proposed, including for ovarian serous cystadenocarcinoma, the most common type of OvCa [199].

High expression of lncRNA TP73-AS1 (TP73 antisense RNA 1) in epithelial OvCa correlates with poor survival, and at the cellular level, stimulates proliferation, invasion, and reduction of apoptosis. Its proposed mechanism of action is binding to the transcriptional repressor EZH2 and recruiting it to the promoter of *CDKN1A* (cyclin-dependent kinase inhibitor 1A, p21), which leads to target silencing [200].

The expression of lncRNA GAS5 is reduced in the case of epithelial OvCa, which correlates with a poor prognosis in patients, as well as with increased cell resistance to cisplatin and related drugs. This lncRNA is able to bind to the transcription factor E2F4, directing it to the *PARP1* gene promoter and weakening the transcription of the latter, which, as a result, decreases the activity of the MAPK pathway and decreases MAPK phosphorylation. An increase in GAS5 expression leads to increased apoptosis and cell cycle arrest at the G0/G1 stage. Compounds such as cycloheximide, rapamycin, and miconazole induce accumulation of GAS5 lncRNA, which may be a potential therapeutic mechanism [194]. In addition, GAS5 also binds to the transcription factor CEBPB, causing a decrease in the expression of the *GDF15* gene and oncosuppression [201]. As a ceRNA, GAS5 also behaves in OvCa as an oncosuppressor (Table 1, [74,75]).

LncRNA UCA1 directly binds in OvCa with AMOT, the YAP regulator. AMOT mediates YAP activation by dephosphorylation and transfer to the nucleus. This results in the activation of YAP target genes related to the Hippo-YAP signaling pathway [202]. Thus, UCA1 both in this mechanism and as a ceRNA (Table 2) acts as an oncogene in OvCa.

LncRNA MALAT1, which in OvCa is a ceRNA for many miRNAs, playing an oncogenic role (Table 2), is also involved in OvCa in another interaction, where it also plays the role of an oncogene. It directly interacts with YAP, suppressing its transition from the nucleus to the cytoplasm. Activation of the transcription of target genes by YAP increases the stemness capacity of OvCa cells [203]. In addition, MALAT1 binds to the Notch1 protein, increasing its activity [204]. It also binds to the alternative splicing factor RBFOX2, decreasing the yield of the alternative proapoptotic isoform KIF1B [205].

LncRNA CACS15 (cancer susceptibility candidate 15) interacts with EZH2 (histone methyltransferase), recruiting it to the *APC* promoter (Wnt pathway regulator), resulting in an increase in the number of H3K27me3 tags in the promoter region. *APC* repression leads to increased proliferation, migration, and invasion of OvCa cells. In clinical practice, an increase in CACS15 expression is correlated with worse patient survival [206].

Other alternative mechanisms of lncRNA action in OvCa are also possible. Thus, lncRNA, acting on the protein mRNA through the mediation of miRNA, may not inhibit miRNA, as in the ceRNA model, but activate it. For example, when analyzing interactions along the MEG3-miR-219a-5p/EGFR (epidermal growth factor receptor) axis in the OvCa, the suppressive lncRNA MEG3 activates the suppressor miR-219a-5p, which inhibits the oncogenic target protein EGFR [207]. Furthermore, the authors showed the participation of MEG3 through this axis in both suppression of EMT and progression of OvCa [201], which is more typical for MEG3.

In addition, the oncogenic lncRNA PVT1 (Table 2), which acts as a ceRNA in many interactions with miRNAs in OvCa, is itself capable of serving as a source of miRNAs, such as miR-1204 and miR-1207 [208]. Moreover, miR-1204 is also oncogenic in OvCa, stimulating glycolysis [209].

Thus, alternative lncRNA mechanisms of influence on the development and progression of OvCa affect in many respects the same range of targets and signaling pathways (p53, HIF-1α, YAP; MAPK and Wnt signaling pathways; glycolysis) as ceRNA-type interactions. One and the same lncRNA, e.g., GAS5, DANCR, MALAT1, PVT1, or UCA1, often acts in OvCa both in the role of ceRNA and in the role of binding a certain protein/mRNA, and the oncogenic or oncosuppressive nature of their influence remains.

## 10. Effect of lncRNA on Signaling Pathways in OvCa Development, Metastasis, and Resistance to Therapy

To date, not only experimental but also generalizing works devoted to the effect of lncRNAs on certain signaling pathways that are significant in OvCa have appeared. For example, Reference [46] indicates lncRNAs affecting the Wnt signaling pathway, such as HOTAIR, SNHG20, HOXD-AS1, CCAT2, MALAT, AWPPH, and HOXB-AS3. However, a general picture of the involvement of lncRNAs in key signaling pathways in OvCa is not considered.

We have reviewed most of the works devoted to the role of lncRNAs in OvCa in different mechanisms, and in Table 3, we have cited and logically organized them as follows. Firstly, those in which lncRNAs themselves, according to the authors, are involved in the regulation of a certain signaling pathway; secondly, those in which the target protein, the expression of which is influenced by lncRNA by one mechanism or another, is indicated in the Panther and/or KEGG (Kyoto Encyclopedia of Genes and Genomes) databases [210,211] as being involved in a certain signaling pathway. LncRNAs, attributed with conflicting data in OvCa, are not included in Table 3.

A systematic analysis based on data from both the literature and the Panther or KEGG databases found that a significant part of lncRNAs somehow influences the key pathways of development and metastasis of OvCa. Moreover, both oncosuppressive and oncogenic lncRNAs affect almost the entire set of key pathways. In addition, proteins, the expression of which is directly or indirectly influenced by lncRNAs, are also associated with many pathways at once, which further emphasizes the importance of lncRNAs in the regulation of biochemical processes in OvCa. Thus, for example, oncosuppressive lncRNA, acting by the ceRNA model, with their protein-targets, were involved in PI3K/AKT/mTOR, NF-κB, MAPK/ERK, HIF/VEGF, JAK/STAT, P53, Wnt/β-catenin, TGF-β, Hippo, and RAS (Table 3). For example, more than 20 oncogenic lncRNA acting by the ceRNA model, with their protein-targets (CCAT1/WNT1, DANCR/IGF2, DQ786243/CREB1, EMX2OS/AKT3, HAGLROS/mTOR, HOTAIR/PIK3R3, HOTAIR/CCND1, HOTAIR/CCND2, HOXD-AS1/FZD4, HOXD-AS1/PIK3R3, HULC/ITGB1, LEF1-AS1/MCL-1, LINC00511/CDKN1, MALAT1, NEAT1/BCL2, PCAT-1/CCND1, PTAL/FN1, RHPN1-AS1, TDRG1/Bcl-xL, TINCR/FGF2, and TMPO-AS1) are involved in the regulation of PI3K/AKT/mTOR signaling (Table 3).

A separate group of lncRNAs significant for EMT was considered. As already mentioned, EMT is associated with metastasis, as well as the acquisition of resistance by cancer cells to chemotherapy. In the work by Mitra et al. [227], three lncRNAs were selected that most significantly affect the expression of genes associated with EMT in OvCa: DNM3OS, MEG3, and MIAT. Moreover, with increased expression of DNM3OS, worse patient survival was observed. Reference [43] discusses the EMT mechanisms associated with the TGF-β pathway, including those involving lncRNAs. In OvCa, H19, MALAT1, and PTAF are distinguished as similar lncRNAs (the latter two are TGF-β-inducible). However, the list of lncRNAs that stimulate or reverse EMT is not limited to this set in OvCa.

Systemic screening of these lncRNAs and their protein-targets revealed more than 20 lncRNAs/protein-targets, implicated in EMT: ADAMTS9-AS2, GAS5, WDFY3-AS2, CCAT1, DLX6-AS1, DQ786243, FLVCR1-AS1, H19, HOTAIR, HOXD-AS1, LINC00963, MALAT1, MLK7-AS1, NEAT1, OIP5-AS1, PTAF, PTAL, PTAR, and LncRNA-ROR, along with their target-proteins. Thus, in Table 4, we list over 20 lncRNA/protein-targets associated with EMT selected from the literature (PubMed, August–September 2020).

As we can see, the suppression of EMT is primarily associated with the effect of lncRNA on target transcription. Stimulation of EMT is often associated with one way or another of inhibition of E-cadherin expression or with activation of the Hippo pathway through YAP1, as well as with regulation of the PI3K/AKT pathway.

Resistance to chemotherapy is associated with the regulation of the expression of proteins (from Table 1, Table 2, Table 3 and Table 4) belonging to cell transporters (ABCC1 [136] and ABCB1 [176]), directly affecting the EMT transition (ZEB1 [150]), and affecting key pathways associated with the development of OvCa and EMT. An example of the latter is the SIRT1 protein [119], which inhibits the activity of p53 and associated apoptosis. The expression of PARP1 protein is suppressed by the oncosuppressive lncRNA GAS5 due to interaction with the transcription factor E2F4 [194] and is stimulated by the oncogenic lncRNA NEAT1 [153]. The PARP1 protein itself stimulates the activity of the NF-κB pathway. Sp1 [183] regulates the expression of the CTR1 transporter, which is associated with the entry of cisplatin into the cell. In addition, Sp1 is associated with a DNA damage response [228].

## 11. Conclusions

Ovarian cancer develops asymptomatically up to advanced stages, and it is characterized by extensive metastasis, chemoresistance, and poor prognosis. The metastasis in OvCa is mainly peritoneal, with the formation of ascites, and its main feature is the absence of a barrier between the primary tumor and the abdominal cavity. An important role in OvCa metastasis is played by EMT, the transition from an immobile polarized epithelial cell associated with the environment to a mobile cell with mesenchymal morphology.

In our review, we discussed the role of lncRNA in pathogenesis, metastasis, and EMT of OvCa. The role of miRNA as a “master regulator” of signaling cascades in the cell is widely known, including in OvCa. However, the discovery of up to 100 thousand lncRNA molecules possessing various but primarily regulatory functions attracted the attention of researchers and the number of related publications is growing sharply.

The mechanism of action of lncRNAs as endogenous RNAs, competing with mRNAs of protein-coding genes for binding to miRNAs (ceRNA model), is becoming increasingly popular and well-proven for tumors with different localizations, including OvCa. According to the literature data we have collected (PubMed, August–September 2020), this mechanism in OvCa cells is realized with the participation of at least 72 lncRNAs, of which about 58 are oncogenic. Instead, oncosuppressive lncRNAs acting in OvCa like ceRNAs are only 14 so far—4 times less frequent. Whether this is a characteristic feature of lncRNAs or reflects the orientation of researchers towards the search for molecular drivers of oncogenesis is still unclear.

For a number of oncogenic lncRNAs (CCAT1, CDKN2-AS1, HOTAIR, HOXD-AS1, MALAT1, NEAT1, PVT1, TUG1, etc.) and some suppressive lncRNAs, several lncRNA/miRNA/mRNA axes have been found (see Figure 3, Figure 4, Figure 5, Figure 6 and Figure 7, Table 1 and Table 2), which reveals different functions of lncRNAs in OvCa for each and confirms the presence of several miRNA response elements in these lncRNAs. Therefore, the interaction and competition among different types of endogenous RNAs, including mRNAs, miRNAs, and lncRNAs, is a newly proposed form of gene regulation that plays an important role in the pathogenesis and metastasis of OvCa.

In addition to the ceRNA mechanism mediated by miRNA, alternative mechanisms of the direct action of lncRNA on mRNA or protein are widespread, examples of which were also considered in our review. Moreover, we revealed some lncRNAs (DANCR, GAS5, MALAT1, UCA1, etc.) acting through both modes: according to the ceRNA model and without miRNA mediation, but regulating different targets. It should be emphasized that we have included only the results, obtained and checked quite convincingly with proven methods, such as the dual-luciferase test, pull-down assay, RIP assay, etc.

The wide possibilities of the influence of lncRNA on the reversibility and dynamics of processes in the cell by competitive interactions are of interest. The ambiguity of miRNA action is well known—depending on the cellular context, they can exhibit both oncogenic and suppressive properties [32]. A duality of behavior was also found for some lncRNAs, as in the case of MEG3 (Table 1, Figure 3) or TTN-AS1 (Table 2). We suggest that such dualism may depend on the properties of the targets and their feedback on lncRNA through a feedback loop.

Our systematic analysis, based on literature data and the Panther or KEGG databases, shows that a significant part of lncRNAs influences the key pathways of OvCa metastasis and EMT. Moreover, both oncosuppressive and oncogenic lncRNAs affect almost the entire set of key pathways. In addition, proteins, the expression of which is directly or indirectly influenced by lncRNAs, are also associated with many pathways at once (see Table 3). Systemic screening of lncRNAs and their protein-targets revealed more than 20 lncRNAs, entered into EMT, e.g., ADAMTS9-AS2, GAS5, WDFY3-AS2, CCAT1, H19, HOTAIR, HOXD-AS1, MALAT1, NEAT1, OIP5-AS1, PTAF, PTAL, PTAR, etc., along with their protein-targets (see Table 4). Therefore, suppression of EMT is associated with the effect of lncRNAs on target transcription.

More than 30 lncRNAs, acting by the ceRNA mechanism, turned out to be predictors of the prognosis of survival and/or factors of response to therapy in patients with OvCa. For example, among suppressive ones such as GAS5, LINC01125, and XIST, we see improved prognosis and/or increased chemosensitivity (see Table 1). On the contrary, among oncogenic lncRNAs, acting by the ceRNA model, e.g., CCAT1, CDKN2B-AS1, EMX2OS, GIHCG, HAGLROS, HAS2-AS1, HOTAIR, HOXD-AS1, KCNQ1OT1, and many others, we can see decreased survival and/or increased chemoresistance (see Table 2).

To date, some detailed reviews have collected data on lncRNAs in OvCa as markers of the disease, predictors of survival, as well as on lncRNAs and drug resistance, mainly Reference [22] and an earlier review [229]. Here, we focused primarily on the mechanisms of carcinogenesis and metastasis, including those significant from the point of view of drug resistance.

## Figures and Tables

**Table 1:** Axes of suppressor lncRNAs and their role in ovarian cancer progression.

Axis lncRNA/miRNA/mRNA	LncRNA Expression; Involvement in Suppression of Progression, Influence on Prognosis, Survival, Drug Sensitivity	References
ADAMTS9-AS2/miR-182-5p/FOXF2	downregulated; reduced invasion, EMT in vitro, in vivo	[72]
EPB41L4A-AS2/miR-103a/RUNX1T1	inhibited migration, invasion in vitro, in vivo	[73]
GAS5/miR-21/SPRY2	suppressed migration, invasion in vitro, better prognosis	[74]
GAS5/miR-196a-5p/HOXA5	downregulated; suppressed proliferation, EMT, migration	[75]
HAND2-AS1/miR-340-5p/BCL2L11	downregulated, hypermethylated; inhibits migration, invasion, adhesion to extracellular matrix in vitro, in vivo	[68,76]
HOTAIRM1/miR-106a-5p/ARHGAP24	downregulated; inhibited migration, invasion, metastasis	[77]
LINC01088/miR-24-1-5p/PAK4	under-expressed; inhibited proliferation, xenografts	[78]
LINC01125/miR-1972	reduced proliferation, enhanced cisplatin sensitivity	[79]
LINC01133/miR-205/LRRK2	suppresses invasion, migration in vitro, in vivo	[80]
MAGI2-AS3/miR-15-5p (miR-374a-5p, miR-374b-5p)/PTEN	downregulated, hypermethylated; decreased migration, viability, adhesion to extra cellular matrix	[81]
MAGI2-AS3/miR-525-5p/MXD1	inhibited cell cycle, migration, invasion; MYC signaling	[69]
MIR503HG/miR-31-5p	downregulated; inhibited invasion, migration; methylation of miR-31 gene by MIR503HG	[82]
MORT/miR-21	downregulated; inhibited proliferation, in vitro	[83]
WDFY3-AS2/miR-18a/RORA	suppressed migration, invasion, EMT, in vitro, in vivo	[84]
XIST/miR-214-3p/PTEN	downregulated; inhibited migration, invasion, in vitro, in vivo, xenografts, increase chemo-sensitivity	[85,86]
Suppressor lncRNA with dual functions		
MEG3/miR-421/PDGFRA	upregulated; promoted angiogenesis, invasion	[87]
MEG3/miR-205-5p/PTEN	downregulated; inhibited migration, invasion, increase cisplatin sensitivity	[88,89]

Note: lncRNA, long non-coding RNA; EMT, epithelial-mesenchymal transition; ADAMTS9-AS2, ADAM metallopeptidase with thrombospondin type 1 motif 9 (ADAMTS9) antisense RNA 2; FOXF2, forkhead-related transcription factor 2; EPB41L4A-AS2, erythrocyte membrane protein band 4.1 like 4A (EPB41L4A) antisense RNA 2; RUNX1T1, runt-related transcription factor 1 (RUNX1) partner transcriptional co-repressor 1; GAS5, growth arrest-specific 5; SPRY2, sprouty homolog 2; HOXA5, homeobox A5; HAND2-AS1, heart and neural crest derivatives expressed 2 (HAND2) antisense RNA 1; BCL2L11, BCL2 like 11 (apoptosis facilitator); HOTAIRM1, HOXA transcript antisense RNA, myeloid-specific 1; ARHGAP24, Rho GTPase activating protein 24; PAK4, p21-activated kinase 4; LRRK2, leucine rich repeat kinase 2; MAGI2-AS3, membrane-associated guanylate kinase, WW and PDZ domain-containing 2 (MAGI2) antisense RNA 3; PTEN, phosphatase and tensin homolog; MXD1, MYC-associated factor X (MAX) dimerization protein 1; MORT, mortal obligate RNA transcript; WDFY3-AS2, WD repeat and FYVE domain-containing 3 (WDFY3) antisense RNA 2; RORA, RAR-related orphan receptor A; XIST, X inactive specific transcript; MEG3, maternally expressed 3; PDGFRA, platelet-derived growth factor receptor alpha; One of the official synonymous names of the lncRNA/mRNA from GeneCards [90] is provided, which gives the best idea of its function.

**Table 2:** Axes of oncogenic lncRNAs acting as ceRNAs and their role in ovarian cancer.

Axis lncRNA/miRNA/mRNA	LncRNA Expression; Involvement in Progression, Influence on Prognosis, Survival, Drug Resistance, Signaling Pathways	Reference
AB209371/miR-203/BIRC5	upregulated; advanced clinical stages	[92]
LncRNA-ATB/miR-204-3p/NID1	tumorigenesis in vitro, in vivo, invasion	[93]
CASC9/miR-758-3p/LIN7A	proliferation, migration, invasion, in vitro, in vivo	[94]
CCAT1/miR-152/ADAM17 (WNT1) CCAT1/miR-130b/STAT3 (ZEB1)	upregulated; EMT, migration, invasion, metastasis, FIGO stage, poor survival	[95]
CCAT1/miR-1290	tumor size, metastasis, prognosis	[96]
CCAT1/miR-490-3p/TGFBR1	migration, invasion, EMT, metastasis	[97]
CCAT1/miR-454/BIRC5	in vivo tumor formation, cisplatin resistance	[98]
CCAT2/miR-424	upregulated; proliferation, progression	[99]
CDKN2B-AS1/miR-411-3p/HIF1A	migration, invasion, metastasis, HIF-1α/VEGF/P38	[100]
CDKN2B-AS1/miR-143-3p/SMAD3	migration, invasion, in vivo, poor prognosis	[101]
DANCR/miR-145/VEGF	invasion, angiogenesis, tube formation	[102]
DLEU1/miR-490-3p/CDK1	upregulated; migration, invasion, in vivo	[103]
DLX6-AS1/miR-195-5p/FHL2	upregulated; migration, invasion, EMT	[104]
DQ786243/miR-506/CREB1	migration, invasion, EMT, in vivo, xenograft	[105]
EMX2OS/miR-654/AKT3	invasion, sphere formation, poorer survival, PD-L1	[106]
FLVCR1-AS1/miR-513/YAP1	migration, invasion, EMT, in vivo	[107]
GIHCG/miR-429	promoted cell cycle, colony formation, shorter OS	[108]
H19/miR-370-3p	upregulated; promotes TGF-β-induced EMT	[109]
H19/miR-324-5p/PKM2	promotes aerobic glycolysis (Warburg effect)	[110]
HAGLROS/miR-100/mTOR (ZNRF2)	upregulated; poor prognosis, mTOR pathway	[111]
CREB1-HAS2-AS1/miR-466/RUNX2	invasion, tumor growth in vivo, poor outcome	[112]
HOST2/let-7b	upregulated; migration, invasion, metastasis	[113]
HOTAIR/miR-1, miR-214-3p, miR-330-5p/MAPK1	upregulated; migration, invasion	[114]
HOTAIR/miR-214, miR-217/PIK3R3	upregulated; proliferation, migration, invasion	[115]
HOTAIR/miR-373/Rab22a	upregulated; migration, invasion, metastasis	[116]
HOTAIR/miR-206/CCND1 (CCND2)	upregulated; migration, invasion, metastasis	[117]
HOTAIR/miR-200c/SNAIL	EMT, migration, invasion, tumorigenicity in vivo	[118]
HOTAIR/miR-138-5p/EZH2, SIRT1	upregulated; promoted cisplatin resistance	[119]
HOTAIR/miR-206/TBX3	upregulated; cell stemness, cisplatin resistance	[120]
HOXD-AS1/miR-133a-3p	EMT, invasion, metastasis, poor OS, Wnt/β-catenin	[121]
HOXD-AS1/miR-608/FZD4	upregulated; migration, invasion, poor prognosis	[122]
HOXD-AS1/miR-186-5p/PIK3R3	migration, invasion, EMT, poor PFS/OS, PIK3R3	[123]
HULC/miR-125a-3p	migration, invasion, PI3K/AKT/mTOR pathway	[124]
KCNQ1OT1/miR-212-3p/LCN2	migration, invasion, in vitro, in vivo, shorter OS	[125]
KCNQ1OT1/miR-142-5p/CAPN10	upregulated; migration in vitro, poor OS	[126]
LEF1-AS1/miR-1285-3p	migration, invasion, metastasis, poor prognosis	[127]
LINC00152/miR-125b/MCL-1	upregulated; grade, clinical stage, poor prognosis	[128]
LINC00161/miR-128/MAPK1	xenograft tumor model in vivo, drug resistance	[129]
LINC00319/miR-423-5p/NACC1	upregulated; proliferation, migration, invasion	[130]
LINC00339/miR-148a-3p/ROCK1	upregulated; migration, invasion, poor prognosis	[131]
LINC00460/miR-338-3p	migration, invasion, metastasis, shorter OS	[132]
LINC00504/miR-1244/PKM2 (HK2, PDK1)	upregulated; aerobic glycolysis/Warburg effect	[133]
ESR1-LINC00511/miR-424, miR-370	proliferation, invasion, poor prognosis, risk model	[134]
LINC00963/miR-378g/CHI3L1	upregulated; migration, EMT	[135]
LINC01118/miR-134/ABCC1	migration, invasion, paclitaxel resistance	[136]
LUCAT1/miR-612/HOXA13	upregulated; metastasis, poorer prognosis	[137]
LUCAT1/miR-199a-5p	upregulated; proliferation, colony formation	[138]
MALAT1/miR-506/iASPP	upregulated; proliferation, DNA synthesis in vitro	[139]
MALAT1/miR-200c	migration, invasion, metastasis, worse prognosis	[140]
MALAT1/miR-143-3p/CMPK	cell viability, migration, invasion, OS/PFS	[141]
MALAT1/miR-211/PHF19	proliferation, migration, xenograft growth	[142]
MALAT1/miR-503-5p	proliferation, JAK2/STAT3 pathway	[143]
MIF-AS1/miR-31-5p/PLCB1	elevated; migratory, invasive abilities of cells	[144]
MLK7-AS1/miR-375/YAP1	invasion, metastasis, EMT, in vitro, in vivo	[145]
MIR4435-2HG/miR-128-3p/CDK14	upregulated; migration, invasion	[146]
NCK1-AS1/miR-137/NCK1	upregulated; migration, invasion, chemo-resistance	[147]
Hur>NEAT1/miR-124-3p	upregulated; migration, invasion, stage, metastasis	[148]
NEAT1/miR-34a-5p/BCL2	increases cells in S phase, suppresses apoptosis	[149]
NEAT1/miR-194/ZEB1	upregulated; paclitaxel resistance in vitro, in vivo	[150]
NEAT1/miR-382-3p/ROCK1	upregulated; migration, invasion, metastasis	[151]
LIN28B>NEAT1/miR-506	migration, invasion in vitro, in vivo, poor prognosis	[152]
NEAT1/miR-770-5p/PARP1	upregulated; cisplatin resistance in vitro, in vivo	[153]
NORAD/miR-155-5p	upregulated; chemo-resistance, xenograft growth	[154]
NORAD/miR-199a-3p	proliferation, migration, invasion, EMT	[155]
Lnc-OC1/miR-34a, miR-34c	migration, invasion, in vitro, in vivo, prognosis	[156]
OIP5-AS1/miR-324-3p/NFIB	upregulated; cell viability, migration, invasion	[157]
OIP5-AS1/miR-137/ZNF217	migration, invasion, EMT in vitro, in vivo	[158]
PCAT-1/miR-129-5p	upregulated; proliferation, inhibits apoptosis	[159]
PCAT-1/miR-124-3p	migration, invasion, Wnt/β-catenin, AKT/mTOR	[160]
PTAF(LINC00922)/miR-25/SNAI2	TGF-β-induced EMT, invasion, metastasis	[161]
PTAR (AP000695.4)/miR-101/ZEB1	migration, EMT, metastasis, in vitro, in vivo	[162]
PTAL/miR-101/FN1	upregulated; EMT, invasion, metastasis	[163]
PVT1/miR-133a	proliferation, migration, invasion, worse PFS/OS	[164]
PVT1/miR-214	invasion, EMT, short PFS/OS, PI3K/AKT	[165]
FOXO4/PVT1/miR-140	upregulated; metastasis, poor survival outcomes	[166]
PVT1/miR-543/SERPINI1	migration, invasion, lower 5-year OS	[167]
RHPN1-AS1/miR-596/LETM1	upregulated; metastasis, DFS/OS, FAK/PI3K/AKT	[70]
RHPN1-AS1/miR-1299	upregulated; migration, invasion, poor prognosis	[71]
LncRNA-ROR/miR-145/FLNB	upregulated; migration and invasion, EMT	[168]
SCAMP1/miR-137/CXCL12	upregulated; invasion, angiogenesis	[169]
SDHAP1/miR-4465/EIF4G2	upregulation; paclitaxel resistance	[170]
SNHG12/miR-129/SOX4	upregulated; migration, metastasis, stage III-IV	[171]
TDRG1/miR-93/RhoC	upregulated; migration, invasion	[172]
TINCR/miR-335/FGF2	tumor size, FIGO stage, lymphatic metastasis	[173]
TMPO-AS1/miR-200c/TMEFF2	EMT, invasion, 5-FU resistance, PI3K/AKT	[174]
TTN-AS1/miR-139-5p/ROCK2	migration, invasion, in vivo, metastasis, poor OS	[175]
TTN-AS1/miR-15b-5p/FBXW7 *	inhibits proliferation, promotes apoptosis	[176]
TUG1/miR-29b-3p/MDM2	migration, invasion in vitro, tumor growth in vivo	[177]
TUG1/miR-29b-3p	metastasis, autophagy, paclitaxel resistance	[178]
TUG1/miR-186-5p/ZEB1	proliferation, invasion, stemness	[179]
TUG1/miR-1299/NOTCH3	upregulated; proliferation, a feedback loop	[180]
UCA1/miR-129/ABCB1	upregulated; proliferation, paclitaxel resistance	[181]
UCA1/miR-654-5p/SIK2	migration, invasion, paclitaxel resistance	[182]
ZFAS1/miR-150-5p/Sp1	proliferation, migration, chemoresistance	[183]

Note: * alternative suppressor function of TTN-AS1; ceRNA, competitive endogenous RNA; EMT, epithelial-mesenchymal transition; DFS, disease-free survival; OS, overall survival; PFS, progression-free survival.

**Table 3:** Involvement of oncosuppressive and oncogenic lncRNAs in critical signaling pathways in ovarian cancer.

Pathway	LncRNAs and Target Proteins *	References
Oncosuppressive lncRNAs		
PI3K/AKT/mTOR	GAS5 (CCND1+), GAS5 (CDKN1A+), HAND2-AS1 (BCL2L11+), MAGI2-AS3 (PTEN+), MAGI2-AS3 (MYC+)	[212], [68], [76], [81], [69]
NF-κB	GAS5 (PARP1+)	[194]
MAPK/ERK	GAS5+, MAGI2-AS3 (MYC+)	[194], [69]
HIF/VEGF	GAS5 (CDKN1A+), MAGI2-AS3 (PTEN+)	[212], [81]
JAK/STAT	GAS5 (CDKN1A+), MAGI2-AS3 (MYC+)	[212], [69]
P53	GAS5 (APAF1+), GAS5 (CDKN1A+), GAS5 (BAX+), MAGI2-AS3 (PTEN+), MAGI2-AS3 (MYC+)	[212], [212], [213], [81], [69]
Wnt/β-catenin	GAS5 (CCND1+), MAGI2-AS3 (MYC+)	[212], [69]
TGF-β	GAS5 (GDF15+), MAGI2-AS3 (MYC+)	[201], [69]
Hippo	MAGI2-AS3 (MYC+)	[69]
RAS	LINC01088 (PAK4+)	[78]
Oncogenic lncRNAs		
PI3K/AKT/mTOR	CCAT1 (WNT1+), DANCR (IGF2+), DQ786243 (CREB1+), EMX2OS (AKT3+), HAGLROS (mTOR+), HOTAIR (PIK3R3+), HOTAIR (CCND1+), HOTAIR (CCND2+), HOXD-AS1 (FZD4+), HOXD-AS1 (PIK3R3+), HULC+, HULC (ITGB1+), LEF1-AS1 (MCL-1+), LINC00511 (CDKN1+), MALAT1+, NEAT1 (BCL2+), PCAT-1 (CCND1+), PTAL (FN1+), RHPN1-AS1+, TDRG1 (Bcl-xL+), TINCR (FGF2+), TMPO-AS1+	[95], [214], [105], [106], [111], [115], [117], [117], [122], [123], [124], [215], [216], [217], [185], [150], [218], [163], [70], [172], [173], [174]
NF-κB	HOTAIR (decreasing Iκ-Bα+), NEAT1 (BCL2+), NEAT (PARP1+), SCAMP1 (CXCL12+), TDRG1 (Bcl-xL+)	[219], [150], [153], [169], [172]
MAPK/ERK	CCAT1 (TGFBR1+), DANCR (IGF2+), H19 (TGF-β+), HOTAIR (MAPK1+), LINC00161 (MAPK1+), MALAT1+, MIR4435-2HG (TGF-β1+), TINCR (FGF2+)	[97], [214], [109], [114], [129], [220], [221], [173]
HIF/VEGF	CCAT1 (STAT3+), CDKN2B-AS1 (HIF1A+), CDKN2B-AS1 (SMAD3+), DANCR (VEGFA+), HOTAIR (MAPK1+), HOTAIR (PIK3R3+), HOXD-AS1 (PIK3R3+), LINC00161 (MAPK1+), LINC00511 (CDKN1+), NEAT1 (BCL2+), NORAD (STAT3+), TDRG1 (P70S6K+)	[95], [100], [101], [102], [114], [115], [123], [129], [217], [150], [222], [172]
JAK/STAT	CCAT1 (STAT3+), HOTAIR (CCND1+), HOTAIR (CCND2+), LINC00511 (CDKN1+), MALAT1+, NEAT1 (BCL2+), NORAD (STAT3+), PCAT-1 (CCND1+), TDRG1 (Bcl-xL+)	[95], [117], [117], [217], [143], [150], [222], [218], [172]
P53	DLEU1 (CDK1+), HOTAIR (PIK3R3+), HOTAIR (CCND1+), HOTAIR (CCND2+). HOTAIR (SIRT1+), HOTAIR (CHEK1+), HOXD-AS1 (PIK3R3+), LINC00511 (CDKN1+), NEAT1 (BCL2+), PCAT-1 (CCND1+), TDRG1 (Bcl-xL+), TUG1 (MDM2+)	[103], [115], [117], [117], [119], [223], [123], [217], [150], [218], [172], [177]
Wnt/β-catenin	CCAT1 (WNT1+), CCAT1 (TGFBR1+), CDKN2B-AS1 (SMAD3+), HOTAIR (CCND1+), HOTAIR (CCND2+), HOXD-AS1+, HOXD-AS1 (FZD4+), KCNQ1OT1+, MALAT1+, MIF-AS1 (PLCB1+), PCAT-1 (CCND1+), PCAT-1+, TTN-AS1 (ROCK2+)	[95], [97], [101], [117], [117], [121], [122], [224], [225], [144], [218], [160], [169]
TGF-β	CCAT1 (TGFBR1+), H19 (TGF-β+), HOTAIR (MAPK1+), LINC00161 (MAPK1+), LINC00339 (ROCK1+), MIR4435-2HG (TGF-β1+), NEAT1 (ROCK1+), TDRG1 (P70S6K+), ZFAS1 (Sp1+)	[97], [109], [114], [129], [131], [221], [151], [172], [183]
Notch	CCAT1 (ADAM17+), DLX6-AS1+, MALAT1 (NOTCH1+), TUG1 (NOTCH3+)	[95], [226], [204], [180]
Hippo	CCAT1 (WNT1+), CCAT1 (TGFBR1+), CCAT1 (BIRC5+), CDKN2B-AS1 (SMAD3+), FLVCR1-AS1 (YAP1+), H19 (TGF-β+), HOTAIR (CCND1+), HOTAIR (CCND2+), HOXD-AS1 (FZD4+), MALAT1 (YAP1+), MLK7-AS1 (YAP1+), MIR4435-2HG (TGF-β1+), PCAT-1 (CCND1+), PTAF (SNAI2+), UCA1 (YAP+)	[95], [97], [98], [101], [107], [109], [117], [117], [122], [203], [145], [221], [218], [161], [202]
RAS	CCAT1 (STAT3+), DANCR (IGF2+), HOTAIR (MAPK1+), LINC00161 (MAPK1+), NEAT1 (BCL2+), NORAD (STAT3+), TDRG1 (RhoC+), TINCR (FGF2+)	[95], [214], [114], [129], [150], [222], [172], [173]

Note: * the + sign marks lncRNAs and target proteins that are themselves directly involved in signaling (according to literature or the Panther and/or KEGG (Kyoto Encyclopedia of Genes and Genomes) databases [210,211]). In each row, the sequence of references corresponds to the sequence of lncRNAs.

**Table 4:** Oncosuppressive and oncogenic lncRNAs associated with epithelial-mesenchymal transition (EMT).

LncRNA	Protein	Reference
Oncosuppressive lncRNAs and protein targets		
ADAMTS9-AS2	FOXF2 (forkhead-related transcription factor 2)	[72]
GAS5	HOXA5 (homeobox protein Hox-A5)	[75]
WDFY3-AS2	RORA (retinoid-related orphan receptor-alpha)	[84]
Oncogenic lncRNAs and protein targets		
CCAT1	ADAM17 (ADAM metallopeptidase domain 17), WNT1, STAT3, ZEB1	[95]
CCAT1	TGFBR1	[97]
DLX6-AS1	FHL2 (downregulated in rhabdomyosarcoma LIM protein)	[104]
DQ786243	CREB1 (active transcription factor CREB)	[105]
FLVCR1-AS1	YAP1	[107]
H19	TGF-β	[109]
HOTAIR	SNAIL	[118]
HOXD-AS1	β-catenin, cyclin D1, c-Myc	[121]
HOXD-AS1	PIK3R3	[123]
LINC00963	CHI3L1 (chitinase 3-like 1)	[135]
MALAT1	KIF1B (kinesin family member 1B)	[205]
MALAT1	YAP1	[203]
MLK7-AS1	YAP1	[145]
NEAT1	ZEB1	[150]
OIP5-AS1	ZNF217 (zinc finger protein 217)	[158]
PTAF	SNAI2 (SNAIL family transcriptional repressor 2)	[161]
PTAR	ZEB1	[162]
PTAL	FN1 (fibronectin 1)	[163]
LncRNA-ROR	FLNB (filamin B)	[168]
TMPO-AS1	TMEFF2 (transmembrane protein with EGF like and two follistatin-like domains)	[174]

## Abbreviations

OvCa	ovarian cancer
ceRNA	competitive endogenous RNA
lncRNA	long non-coding RNA
EMT	epithelial-mesenchymal transition
CCAT1	colon cancer-associated transcript 1
HOTAIR	HOX transcript antisense intergenic RNA
DANCR	differentiation antagonizing non-protein coding RNA
GAS5	growth arrest-specific transcript 5
MALAT1	metastasis-associated lung adenocarcinoma transcript 1
MEG3	maternally expressed 3
NEAT1	nuclear-enriched abundant transcript 1
TUG1	taurine upregulated 1
UCA1	urothelial carcinoma-associated 1
3′-UTR	3′-untranslated region
